# A Bi‐Adjuvant Senolytic Nanovaccine Modulating Dendritic Cell Senescence Ameliorates Aging‐Related Metabolic Dysfunction

**DOI:** 10.1002/advs.78545

**Published:** 2026-10-28

**Authors:** Qingxu Song, Xiaoxue Du, Wenbo Dong, Ying Wang, Xiaojing Wang, Qiliang Yin

**Affiliations:** ^1^ Department of Spine Surgery The First Hospital of Jilin University Changchun People's Republic of China; ^2^ Departments of Laboratory Medicine Lequn Branch The First Hospital of Jilin University Changchun People's Republic of China; ^3^ Medical School of Changchun Sci‐Tech University Changchun People's Republic of China; ^4^ Department of Geriatric Medicine Geriatric Medicine Center The First Hospital of Jilin University Changchun People's Republic of China

**Keywords:** Bi‐adjuvant, dendritic cell, metabolic dysfunction, senescence, senolytic nanovaccine

## Abstract

Senolytic vaccines hold promise for eliminating senescent cells, but their efficacy is limited by poor seno‐antigen immunogenicity and age‐related dendritic cell (DC) dysfunction. Here, we engineered an active biomaterial‐enabled bi‐adjuvant senolytic nanovaccine (CK5‐GC‐NV) by co‐assembling CpG oligodeoxynucleotide with a fusion protein of the seno‐antigen Gpnmb and a cationic spider silk protein variant (CK5). Beyond antigen delivery, the CK5 scaffold acts as an intrinsic senomodulator associated with the downregulation of mitotic kinases Aurka/b and a concomitant decrease in senescence‐associated PI3K/AKT phosphorylation in DCs. This intervention attenuates aged DC phenotypes, restores antigen cross‐presentation, and synergizes with CpG to elicit potent cytotoxic T‐lymphocyte responses against senescent targets. Across multiple aging mouse models, CK5‐GC‐NV immunization orchestrated targeted clearance of Gpnmb^+^ senescent adipocytes, profoundly ameliorating metabolic dysfunction and extending lifespan. This multifunctional biomaterial platform integrates seno‐antigen immunogenicity enhancement with the restoration of aged DC function, providing a promising proof‐of‐concept for antigen‐directed gerotherapeutics.

## Graphical abstract

An active biomaterial‐based bi‐adjuvant nanovaccine restores the immunocompetence of aged dendritic cells to overcome senescent cell tolerance. Accompanied by the downregulation of mitotic kinases Aurka/b and attenuation of senescence‐associated PI3K/AKT phosphorylation, the nanovaccine enhances antigen‐presenting capacity and promotes potent cytotoxic T‐cell‐mediated clearance of Gpnmb‐expressing senescent adipocytes. This strategy effectively ameliorates aging‐associated metabolic dysfunction, providing a promising proof‐of‐concept for biomaterial‐assisted gerotherapeutics.

## 1 Introduction

Senescent cells progressively accumulate in various tissues during organismal aging and in response to metabolic stress, acting as central drivers of age‐related pathologies [1, 2, 3]. While the pharmacological clearance of senescent cells (senolysis) has been shown to alleviate physiological aging and metabolic dysfunction in preclinical models [4, 5], current small‐molecule senolytics targeting anti‐apoptotic pathways often lack tissue specificity and pose substantial risks of off‐target toxicity [6]. The identification of seno‐antigens has enabled senolytic vaccines to achieve antigen‐directed senescent cell clearance with improved safety profiles [7, 8], representing an innovative paradigm in geroscience. However, the development of effective senolytic vaccines is fundamentally challenged by weak senescence‐antigen immunogenicity [9, 10], aging‐impaired DC function [11, 12], and senescent cell‐mediated immunosuppression [13]. Notably, in aged organisms, DC senescence emerges as a critical obstacle to the efficacy of senolytic vaccination [14, 15]. Therefore, an effective immunosenolytic strategy requires precise presentation of seno‐antigens coupled with immune rejuvenation to overcome DC priming deficits and achieve optimal senescent cell elimination.

DCs undergo dynamic, age‐dependent functional changes throughout life and mirror the immunological age of the host [13, 16]. In early life (fetuses/neonates), DCs exhibit immature functionality [17], whereas in advanced age, accumulating evidence indicates that e aged DCs exhibit impaired antigen uptake, defective cross‐presentation, attenuated co‐stimulatory signaling, and reduced capacity to prime T‐cell responses [18, 19]. Such senescence in DCs provides a mechanistic explanation for the poor vaccine responsiveness observed in elderly individuals [20, 21]. Senescent DCs exhibit cell‐intrinsic hyperactivation of the PI3K/AKT pathway, which disrupts antigen cross‐presentation and CD8^+^T‐cell priming against seno‐antigens [11, 22, 23]. These immunological constraints severely undermine the efficacy of current senolytic vaccines, particularly those targeting poorly immunogenic seno‐antigens. Emerging nanotechnological platforms enable the engineering of multifunctional senolytic vaccines that integrate antigen delivery and immune modulation [12, 24, 25, 26]. Nanoarchitectured vaccines can precisely deliver adjuvants to senescent DCs to restore their activation without triggering excessive systemic inflammation, consequently amplifying vaccine‐elicited immunity in aged individuals [12, 27]. Such senolytic vaccines necessitate a highly coordinated assembly to simultaneously enhance antigen immunogenicity and actively modulate DC senescence, thereby driving the efficient cytotoxic T‐lymphocyte activation required for targeted senolysis. In this integrated gerotherapeutic system, the bi‐adjuvant platform operates through two functionally distinct, compartmentalized mechanisms: direct intracellular uptake of the CK5 scaffold by local DCs attenuates their senescence‐associated signaling to rescue antigen‐presenting competence, whereas distant senescent adipocytes undergo extrinsic, targeted elimination orchestrated by vaccine‐activated Gpnmb‐specific CD8^+^ cytotoxic T lymphocytes.

Here, we engineered an active biomaterial‐based bi‐adjuvant senolytic nanovaccine (CK5‐GC‐NV) via the electrostatic co‐assembly of the immunoadjuvant CpG oligodeoxynucleotide (CpG ODN) with a fusion protein, which comprises the seno‐antigen glycoprotein non‐metastatic melanoma protein B (Gpnmb) and a cationic spider silk protein variant (CK5) (Figure 1A). We demonstrate that the CK5 scaffold is not merely an inert carrier, but an active senomodulator that is associated with the downregulation of the mitotic kinases Aurka/b and t the concurrent suppression of senescence‐associated PI3K/AKT phosphorylation in DCs. This mechanistic intervention effectively delays DC senescence, potentiates antigen cross‐presentation, and synergizes with CpG ODN to drive a robust, antigen‐specific cytotoxic T‐lymphocyte response. In high‐fat diet‐induced, D‐galactose‐induced, and naturally aged mouse models, subcutaneous immunization with CK5‐GC‐NV orchestrated the highly efficient elimination of Gpnmb^+^ senescent adipocytes. This targeted clearance successfully alleviated aging‐associated metabolic dysfunction and significantly extended organismal lifespan. Intriguingly, comparable therapeutic effects were achieved using a DC vaccine loaded with the non‐senomodulatory K36‐GC‐NV only when combined with the pharmacological Aurka/b inhibitor Danusertib (Danu). This comparative validation collectively underscores the critical dual role of CK5‐GC‐NV in simultaneously modulating DC senescence and enhancing immunogenicity, offering a highly promising and translatable strategy for precision senotherapeutics against aging‐associated metabolic diseases (Figure 1B).

**FIGURE 1:**
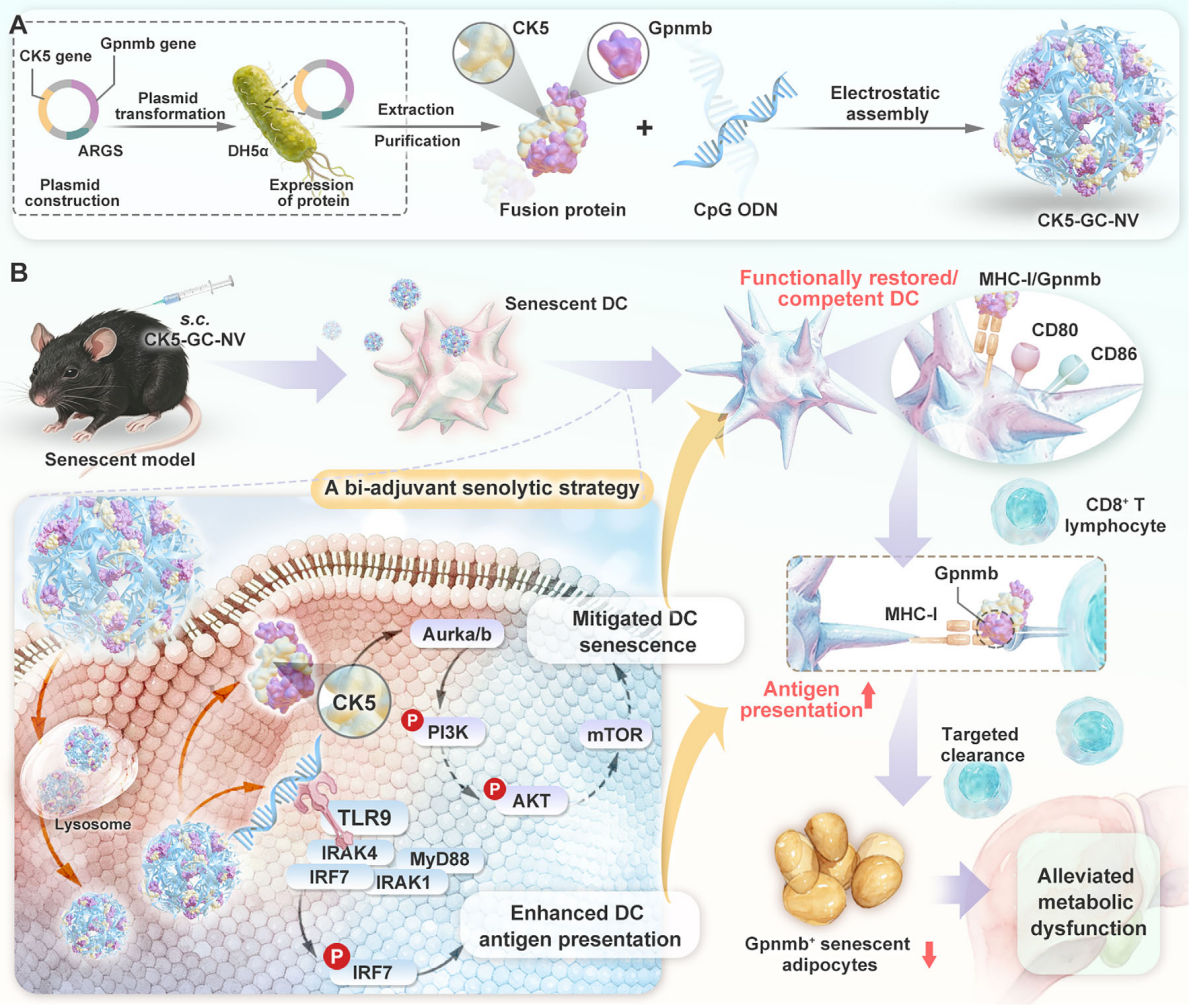
Construction and therapeutic mechanism of the bi‐adjuvant senolytic nanovaccine CK5‐GC‐NV. (A) Schematic illustration of CK5‐GC‐NV fabrication. The fusion protein comprising the multifunctional protein CK5 and the senescence‐associated antigen Gpnmb was recombinantly expressed and purified, followed by electrostatic assembly with CpG oligodeoxynucleotides (CpG ODN) to generate CK5‐GC‐NV. (B) Schematic representation of the mechanism of action of CK5‐GC‐NV in aged mice. Following subcutaneous administration, CK5‐GC‐NV is internalized by senescent dendritic cells (DCs). CpG ODN activates TLR9 signaling to promote DC maturation and antigen presentation, whereas CK5 internalization is accompanied by the downregulation of Aurka/b and attenuation of senescence‐associated PI3K/AKT signaling to alleviate DC senescence and restore DC function. DCs with attenuated senescence and restored competence exhibit enhanced MHC‐I antigen presentation and co‐stimulatory molecule expression, resulting in improved priming of Gpnmb‐specific CD8^+^ T cells. The activated cytotoxic T cells selectively eliminate Gpnmb^+^ senescent adipocytes, thereby reducing senescent cell burden and alleviating metabolic dysfunction.

## 2 Materials and Methods

### 2.1 Gene Expression and Protein Purification

CK5 is a cationic protein with self‐assembly capability. The plasmids encoding CK5 (pET25b‐CK5) (Table S1) were kindly provided by Prof. HaoFan Jin. The Glycoprotein nonmetastatic melanoma protein B (Gpnmb) gene (Table S1) from mice was synthesized by a genomic company (GENEWIZ, the U.S.A.) and inserted into the pET25b vector, and the H‐2K^b^‐restricted Gpnmb nonamer peptide (RRGDGRWKD; Table S2) from mice was synthesized by Sangon Biotech company (Shanghai, China). The plasmids encoding CK5‐Gpnmb were generated in our laboratory. In brief, CK5 and Gpnmb were genetically linked at the gene level via homologous recombination, and the resulting construct was subsequently transfected into competent Escherichia coli DH5α cells (Novagen, USA). Plasmids encoding CK5‐Gpnmb were obtained following the amplification and culture of the competent cells. Finally, the pET25b‐CK5‐Gpnmb (Table S1) expression vectors were transformed into chemically competent E. coli BLR (DE3) cells (Novagen, USA). The protocols for protein production followed the same procedures as previously described [28].

For protein purification, cultured E. coli cells were harvested by centrifugation and resuspended in lysis buffer (50 mm sodium phosphate buffer, pH 7.8, 0.5 mm NaCl, 20 mm imidazole) to an optical density (OD600) of 50. The cells were then disrupted using a constant cell disrupter (Constant Systems Ltd., Daventry, UK). Cell debris was removed by centrifugation, and the proteins were purified from the supernatant under native conditions using Ni‐Sepharose chromatography (General Electric Company). The product‐containing fractions were collected and subsequently dialyzed against 50 mm sodium phosphate buffer, pH 7.8. The proteins were then further purified by cation exchange chromatography using an SP HP column (General Electric Company). The buffers used for this step were buffer A (50 mm sodium phosphate, 50 mm NaCl, pH 7.4) and buffer B (50 mm sodium phosphate, 2 m NaCl, pH 7.4). The endotoxin levels of the purified protein products were assessed using the Limulus amebocyte lysate (LAL) assay (Zhanjiang A&C Biological LTD, China). The results indicated that the residual endotoxin level in the purified protein products was below 0.1 EU/mg (or < 0.05 EU/mL), which was an accepted standard for evaluating endotoxin toxicity. The product‐containing fractions were extensively dialyzed against ultrapure water. Subsequently, the purified protein CK5‐Gpnmb (Table S2) was lyophilized and utilized for subsequent experiments. K36 is likewise a cationic protein with self‐assembly capability. The fusion protein K36‐Gpnmb was obtained using the same procedure described above.

### 2.2 Preparation and Characterization of CK5‐Gpnmb‐CpG

CpG ODN with a fully nuclease‐resistant phosphorothioate backbone was used. The CpG was dissolved in water at a concentration of 2 mg/mL in a round‐bottom flask, to which varying concentrations of CK5‐Gpnmb protein were added gradually. In detail, CK5‐Gpnmb and CpG ODN were electrostatically complexed in PBS (pH 7.4) at mass ratios of 1:2 to 1:6 (w/w) under gentle agitation for 30 min at room temperature. At the end of the reaction, the mixture was centrifuged at 12 000 rpm for 10 min. The hydrodynamic size and zeta potential of the CK5‐Gpnmb‐CpG nanovaccine (CK5‐GC‐NV) were evaluated using dynamic light scattering (DLS) with a Zetasizer Nano ZS instrument (ZEN3600, Malvern Instruments Limited, UK). Additionally, agarose gel electrophoresis was employed to assess the packing capacity of CpG at various charge ratios of CK5‐Gpnmb to CpG; meanwhile, the CK5‐Gpnmb protein was determined by sodium dodecyl sulfate polyacrylamide gel electrophoresis (SDS‐PAGE) on a 12% polyacrylamide gel. Finally, the CK5‐GC‐NV was synthesized at the optimal ratio of CK5‐Gpnmb to CpG. The sizes and morphologies of CK5‐GC‐NV were characterized using transmission electron microscopy (TEM) and scanning electron microscope (SEM) (Hitachi SU‐70). The CK5‐GC‐NV was deposited onto a silicon wafer and dried, followed by visualization using STEM‐based energy dispersive x‐ray spectroscopy (EDS) mapping.

Using the aforementioned approach, the fusion protein K36‐Gpnmb was assembled with CpG to generate the K36‐Gpnmb‐CpG nanovaccine (K36‐GC‐NV), which served as the control vaccine in subsequent experiments.

### 2.3 Acquisition of Bone Marrow‐Derived Dendritic Cells (BMDCs) and Preparation of the DC Vaccine

Bone marrow‐derived dendritic cells (BMDCs) were isolated from the femurs and tibias of female wild‐type C57BL/6 mice, aged 6–8 weeks, under aseptic conditions. The BMDCs were then seeded in 6‐well culture plates at a density of 1 × 10^6^ cells/mL in 2 mL of RPMI 1640 complete medium, supplemented with 10% fetal bovine serum (Inner Mongolia Opcel Biotechnology Co., Ltd), IL‐4 (10 ng/mL) (PeproTech, USA), and GM‐CSF (20 ng/mL) (PeproTech, USA), and incubated in a cell culture incubator. On days 2 and 4, the culture medium was refreshed with new medium containing IL‐4 (10 ng/mL) and GM‐CSF (20 ng/mL). On day 6, the immature BMDCs were harvested for subsequent experiments.

Bone marrow cells harvested from 6–8‐week‐old mice were differentiated into BMDCs, followed by incubation with K36‐GC‐NV for 24 h. The K36‐GC‐NV–loaded BMDCs were then collected, formulated as a DC vaccine, and administered to mice by subcutaneous injection into the inguinal region.

### 2.4 Antigen Phagocytosis, Intracellular Distribution, and Surface Presentation in Dendritic Cells

BMDCs were incubated with Gpnmb‐FITC‐loaded Gpnmb/CpG, K36‐GC‐NV, or CK5‐GC‐NV (at a Gpnmb concentration of 10µg/mL) for 4 h. The cells were then washed with PBS and stained with 4',6‐diamidino‐2‐phenylindole (DAPI) (Beyotime, China) for 5 min. Afterward, phagocytosis of the Gpnmb was observed under a confocal laser scanning microscope (CLSM). For the intracellular distribution analysis, BMDCs were incubated with Gpnmb‐FITC‐loaded Gpnmb/CpG, CK5‐Gpnmb CK5‐GC‐NV, or K36‐GC‐NV (at a Gpnmb concentration of 10µg/mL) for 24 h. Following incubation, the cells were washed with PBS and stained with LysoTracker Deep Red and Hoechst 33342 for nuclear visualization (Beyotime, China) for 25 min. Subsequently, fresh medium was added, and the intracellular localization of Gpnmb was examined using a confocal laser scanning microscope. BMDCs were treated with the indicated formulations for 48 h, washed with PBS, and then incubated with a FITC‐conjugated conformation‐specific antibody against the H‐2K^b^–Gpnmb (RRGDGRWKD) complex (GENEWIZ, USA) for 60 min at 4°C in the dark. According to the manufacturer's quality control and validation procedures, the specificity of this custom antibody was confirmed by comparative ELISA counter‐screening against the cognate H‐2K^b^–Gpnmb complex vs. an irrelevant peptide–MHC complex (H‐2K^b^–OVA_257__−__264_, SIINFEKL), showing robust preferential target binding with negligible cross‐reactivity (Table S3). After washing with PBS, cell nuclei were stained with DAPI (Beyotime, China) for 5 min. Finally, the surface cross‐presentation of the Gpnmb peptide on BMDCs was visualized and acquired using a confocal laser scanning microscope.

### 2.5 Acquisition of Spleen Lymphocytes and Sorting of CD8^+^ T Cells

After euthanizing the mice, the spleens were removed and processed into single‐cell suspensions using mouse lymphocyte isolate (DAKEWEI, China). The cell suspension was passed through a strainer (Thermo Fisher Scientific, USA) twice to eliminate any remaining tissue fragments. The cells were then centrifuged at 3000 rpm for 10 min and resuspended in ACK lysis buffer (Biosharp, China) to lyse erythrocytes. Following a 5‐min incubation, the cells were washed with PBS three times. Finally, the lymphocytes were centrifuged again and resuspended in RPMI 1640 complete medium. Spleen lymphocytes were resuspended in sorting buffer. Next, 100µL of the cell suspension (1 × 10^7^ cells) was placed in flow cytometry tubes, and 2µL of Biotin Antibody Mix (Beaver, China) was added. The mixture was incubated at 4°C for 10 min. Following this, 20µL of BeaverBeads (Beaver, China) and Streptavidin (Beaver, China) were added to the tubes, and the contents were mixed thoroughly before incubating again at 4°C for another 10 min. Then, 2.5ml of sorting buffer was added to the flow cytometry tubes. The tubes containing the cells were placed on a magnetic rack for 5 min. Afterward, the cell suspension was carefully transferred into a sterile centrifuge tube containing purified mouse CD8^+^T cells. Finally, the CD8^+^ T cells were collected after centrifugation.

### 2.6 In vitro Cytotoxicity Assay of CD8^+^ T Cells

To evaluate the cytolytic activity of vaccine‐elicited CD8^+^ T cells against Gpnmb‐expressing senescent cells, an in vitro surrogate target cell model was established using the mouse 3T3‐L1 preadipocyte cell line. Briefly, 3T3‐L1 cells were cultured and treated with D‐galactose to induce cellular senescence. The senescent phenotype and the high expression of the target antigen Gpnmb on these cells were subsequently validated. For the co‐culture assay, activated CD8^+^ T cells (effector cells) were sorted from the lymph nodes of immunized mice in different immunization groups using magnetic bead separation as described above. The isolated CD8^+^ T cells were then co‐cultured with the senescent, Gpnmb‐high 3T3‐L1 cells (target cells) at predefined effector‐to‐target (E: T) ratios in 96‐well plates. After a co‐culture period, the specific killing of the target cells was quantitatively assessed by measuring the release of lactate dehydrogenase (LDH) into the culture supernatant using a commercial LDH Cytotoxicity Assay Kit according to the manufacturer's instructions. The percentage of specific lysis was calculated based on the experimental LDH release relative to the maximal and spontaneous release controls.

### 2.7 Cellular Energy Metabolism Studies

Cellular extracellular acidification rate (ECAR) and oxygen consumption rate (OCR) were quantified using an XF96 Extracellular Flux Analyzer (Seahorse Bioscience, North Billerica, MA, USA) in accordance with the manufacturer's protocol. Prior to measurement, BMDCs were plated at an optimized density of 5 × 10^4^ cells per well in a Seahorse XF96 cell culture microplate, which generated robust basal metabolic readouts well above background thresholds (basal OCR > 80pmol/min vs. background < 10pmol/min; basal ECAR > 15 mpH/min vs. background < 2 mpH/min). Each condition was assessed with four to five technical replicates. Following the assay, normalization was carried out immediately using the CellTiter‐Glo Luminescent Cell Viability Assay (Promega, USA) to control for well‐to‐well cell number variations. Briefly, 100µL of CellTiter‐Glo reagent was added to each well, mixed on an orbital shaker for 2 min, and incubated at room temperature for 10 min before reading luminescence on a microplate reader. All raw OCR and ECAR values were normalized to the relative luminescent signals of each well.

### 2.8 Isolation of Adipocytes

After euthanizing the mice, Epididymal visceral adipose tissue (eVAT) was collected and finely minced. The minced tissue was then placed in a composite enzyme digestion solution comprising Collagenase I (1mg/mL), Collagenase II (0.5mg/mL), Collagenase IV (0.5mg/mL), and Trypsin (0.25mg/mL) in a 1:10 ratio, followed by incubation at 37°C for 40 min. After digestion, the reaction was stopped by adding DMEM supplemented with 10% FBS, and the mixture was centrifuged at 400g for 5 min. The resulting pellet was resuspended in PBS for subsequent flow cytometry analysis.

### 2.9 Animals Immune Model

According to previous studies, a high‐fat diet (HFD) can induce adipose tissue senescence in mice and lead to the development of senescence‐associated pathological phenotypes [7, 8]. In this study, C57BL/6 mice were obtained from Changsheng Company (Shenyang, China). In the HFD‐induced aging mouse model, mice were placed on an HFD starting at 4 weeks of age. Mice fed an HFD were subjected to subcutaneous immunization in the inguinal region at week 60. Immunological analysis was conducted at 63 weeks, followed by efficacy analysis at 70 weeks. In a D‐galactose–induced aging model, 8‐week‐old C57BL/6 mice were administered D‐galactose at a dose of 100 mg/kg via intraperitoneal injection every other day, followed by inguinal subcutaneous immunization at 24 weeks of age and efficacy assessment at 30 weeks of age. In the natural aging mouse model, mice maintained on a standard diet received inguinal subcutaneous immunization at 75 weeks of age. Immunological analysis was conducted at 78 weeks, followed by efficacy analysis at 85 weeks. All mice were housed under specific pathogen–free (SPF) conditions. All animal experiments were conducted in accordance with the ARRIVE guidelines and approved by the Animal Ethics Committee of the First Hospital of Jilin University (Approval Number: 20260225‐121). Female C57BL/6 mice were randomized into experimental cohorts using computer‐generated random allocation. Investigators performing histological evaluations (e.g., adipocyte cross‐sectional area quantification) and flow cytometric analysis were blinded to group allocations through the assignment of coded specimen numbers. Humane endpoints were established prior to study initiation: animals displaying severe lethargy, inability to access food or water, or weight loss exceeding 20% of peak body weight were humanely euthanized by carbon dioxide inhalation followed by cervical dislocation and recorded as non‐censored events. No unscheduled animal dropouts occurred prior to defined efficacy or survival timelines.

### 2.10 Flow Cytometry

Cell surface flow cytometry staining: The single‐cell suspension from different treatment groups was washed three times with PBS and then stained with fluorescence‐labeled antibodies at 4°C for 30 min. Following centrifugation, the cells were washed twice with PBS before being analyzed by flow cytometry. Intracellular flow cytometry staining: Following surface antigen staining, cells were fixed with Fixation Buffer (BioLegend, USA) for 20 min at room temperature in the dark. After centrifugation (350 × g, 5 min), cells were permeabilized with Intracellular Staining Perm Wash Buffer (BD FACS CantoII, USA), incubated with fluorochrome‐conjugated intracellular antibodies (e.g., IFN‐γ, p16) at 4°C for 30 min in the dark, washed twice with PBS, and analyzed by flow cytometry. The flow cytometry panel included the following antibodies from BioLegend (USA): APC‐CD11c, FITC‐CD80, PE‐CD86, FITC‐CD8, PE‐CD69, APC‐CD25, PE‐IFNγ, APC‐Granzyme B(GzmB), PE‐PDL1, PE‐CD44, APC‐CD62L, PE‐CD31, and p16‐PE (BD Biosciences). For memory T‐cell subset analysis, viable singlet lymphocytes were gated on the CD8^+^ population; central memory T cells (Tcm) were identified as CD8^+^CD44^+^CD62L^+^, and effector memory T cells (Tem) were defined as CD8^+^CD44^+^CD62L^−^. Gating boundaries were established based on fluorescence minus one (FMO) control. Additionally, the eFluor 660‐Gpnmb antibody (Thermo Fisher Scientific, USA) was excited by APC. Detailed antibody clones, catalog numbers, and working dilutions are cataloged in Table S3. Data acquisition was performed using a BD FACS Canto II flow cytometer.

### 2.11 Enzyme‐Linked Immunosorbent Assay (ELISA)

The supernatant was collected at various time points from the CK5‐GC‐NV in environments with pH 5.6 and pH 7.4. Subsequently, the supernatant was analyzed for Gpnmb levels using a Gpnmb ELISA kit (Mlbio, Shanghai) according to the manufacturer's instructions. The levels of IL‐12p70 secretion in the culture supernatants of BMDCs treated with different formulations were determined using an IL‐12p70 ELISA kit (Mlbio, Shanghai). Similarly, serum samples from various groups of mice were analyzed for circulating levels of representative pro‐inflammatory SASP factors (IL‐6, TNF‐α, and IL‐1β) using specific commercial ELISA kits (Mlbio, Shanghai) in accordance with the manual instructions.

### 2.12 Histological Staining Analyses

Tissue samples from mice were collected and fixed overnight in 10% formalin. Following fixation, the samples were processed and embedded in paraffin. Paraffin sections were then prepared and subjected to hematoxylin and eosin (HE) staining using standard procedures. For the HE staining of adipose tissue, the cross‐sectional areas of adipocytes were measured using an image analyzer (Keyence).

### 2.13 Senescence‐Associated (SA)‐β‐Galactosidase (gal) Assay

Visceral adipose tissue was harvested from mice induced by a high‐fat diet and sectioned into slices approximately 1 mm thick. The tissue slices were fixed at room temperature for 30 min, followed by incubation overnight at 37°C with freshly prepared SA‐β‐gal staining solution (C0602, Beyotime, Shanghai, China) in accordance with the manufacturer's instructions. After staining, the adipose tissue was photographed, and the images were analyzed using ImageJ software.

### 2.14 Western Blot Analysis

Adipose tissue or whole‐cell lysates were prepared using RIPA lysis buffer supplemented with protease and phosphatase inhibitor cocktails (Roche). Total protein concentrations were quantified using a bicinchoninic acid (BCA) protein assay kit (Beyotime, China). Approximately 40–50 µg of the lysates were resolved by SDS–polyacrylamide gel electrophoresis and subsequently transferred onto PVDF membranes (Millipore, USA). After blocking with 5% non‐fat milk or 5% bovine serum albumin (BSA) in TBST for 1 h at room temperature, the membranes were incubated with primary antibodies overnight at 4°C, followed by incubation with horseradish peroxidase‐conjugated secondary antibodies (Jackson, USA) for 1 h at room temperature. Specific protein bands were detected using enhanced chemiluminescence (Amersham, Sweden). The primary antibodies used included Gpnmb, P53, P21, P16, GAPDH, and β‐actin (Proteintech, USA), as well as Aurka, Aurkb, TLR9, MyD88, p‐IRF7, PI3K, p‐PI3K, AKT, p‐AKT(S473), and p‐AKT(T308) (Abcam, UK). Peroxidase‐conjugated AffiniPure goat anti‐mouse IgG (light chain specific) (Proteintech, USA) was used as the secondary antibody at a dilution of 1:2000. Chemiluminescent signals were acquired using a Tanon 4600 imaging system (China). All Western blot experiments were independently performed with at least three biological replicates (*n* = 3). Densitometric quantification of band intensities was conducted using ImageJ software (NIH, USA). Target protein levels were normalized to their corresponding loading controls (GAPDH or β‐actin), while the activation of signaling pathways was determined by calculating the ratio of phosphorylated protein to the corresponding total protein (e.g., p‐AKT/total AKT and p‐PI3K/total PI3K). Quantitative data are presented as relative fold changes. Comprehensive catalog information is summarized in Table S3.

### 2.15 Assessment of Systemic Metabolic Parameters

Mice were housed individually for one week prior to the experiments. Systemic metabolic parameters, including body weight and food intake, were continuously monitored on a weekly basis post‐immunization across both the high‐fat diet (HFD) and natural aging models. Average weekly food consumption per group was calculated by measuring the weekly difference in chow weight in the cage dispensers. On the day of the glucose tolerance test (GTT), the mice were fasted for 6 h before receiving an intraperitoneal injection of glucose at a dose of 1 g/kg body weight in the early afternoon. For the insulin tolerance test (ITT), mice were administered human insulin intraperitoneally at a dose of 1 U/kg body weight. Blood samples were collected from the tail vein at 0, 15, 30, 60, and 120 min post‐administration of each agent, and blood glucose levels were measured using a glucose analyzer (Sanwa Kagaku Kenkyusho).

### 2.16 Statistical Analysis

Statistical analyses were performed using GraphPad Prism 8.0 and FlowJo v10. Data are presented as mean ± standard deviation (SD) with individual biological replicates plotted as discrete data points in all quantitative bar and dot plots. Exact sample sizes (n) representing independent biological experiments or individual animals are specified in the respective figure legends. Two‐group comparisons were evaluated using an unpaired, two‐tailed Student's t‐test, whereas multi‐group comparisons were assessed via one‐way or two‐way ANOVA followed by Tukey's post hoc test. Survival trajectories were evaluated using the Kaplan–Meier method; statistical significance was assessed using the log‐rank (Mantel–Cox) test, and effect sizes were determined by calculating hazard ratios (HR) with 95% confidence intervals (95% CI) using the Mantel–Haenszel approach. A value of P < 0.05 was considered statistically significant. For the glucose tolerance test (GTT) and insulin tolerance test (ITT), blood glucose levels at each time point were analyzed using two‐way ANOVA followed by Tukey's multiple‐comparison test. The area under the curve (AUC) was calculated using the trapezoidal rule and analyzed by one‐way ANOVA followed by Tukey's multiple‐comparison test, statistical significance was defined as ^*^ *p* < 0.05, ^**^ *p* < 0.01, ^***^ *p* <0.001 and ^****^ *p* < 0.0001; ^#^ *p* < 0.05; ^##^ *p* < 0.01, ^###^ *p* < 0.001 and ^####^ *p* < 0.0001; ^&&^ *p* < 0.01, ^&&&^ *p* < 0.001 and ^&&&&^ *p* < 0.0001.

## 3 Results

### 3.1 Fabrication and Characterization of CK5‐GC‐NV

The CK5‐GC‐NV was rationally designed through electrostatic self‐assembly of a cationic chimeric protein (CK5‐Gpnmb) with an anionic TLR9 agonist (CpG ODN), forming stable immunogenic nanocomplexes (Figure 1A). The seno‐antigen peptide Gpnmb was genetically fused with CK5 (Figure S1A), a modular protein scaffold containing highly repetitive elastin‐like polypeptide motifs [(GPGGY)_12_‐(VPGKG)_30_], to construct the cationic chimeric protein component (CK5‐Gpnmb) of the nanovaccine. Electrophoretic analysis demonstrated complete CpG ODN complexation at the optimal 1:4 mass ratio (Figure S1B). The engineered nanocomplexes exhibited size homogeneity (<170 nm) across all formulations, with surface charge transitioning from anionic (−35.2 ± 1.5 mV at 1:2) to cationic (+22.6 ± 2.1 mV at 1:6), achieving charge neutrality at the 1:3.8 stoichiometric ratio (Figure 2A). Consequently, the 1:4 mass ratio was selected for optimal CpG ODN loading, yielding CK5‐GC‐NV nanocomplexes with ∼90 nm hydrodynamic diameter (Figure 2B). Elemental mapping and energy‐dispersive spectroscopy (EDS) revealed uniform distribution of C, N, O, S, and P, confirming effective integration of protein‐nucleic acid components (Figure 2C and Figure S1C). Similarly, transmission electron microscopy (TEM) revealed that K36‐GC‐NV forms nanoparticles with an approximate diameter of 80nm (Figure S1D). CK5‐GC‐NV demonstrated excellent biocompatibility, with Cell Counting Kit‐8 (CCK‐8) assays showing >90% cell viability in human fibroblasts at concentrations up to 300µg/mL (Figure S1E). Comparative assessment at 200µg/mL revealed no significant cytotoxicity vs. PBS controls across all formulations (Gpnmb/CpG, CK5‐Gpnmb, K36‐GC‐NV and CK5‐GC‐NV) (Figure 2D), establishing this concentration for subsequent studies. Consistent with this design, ELISA‐based quantification revealed pronounced pH‐dependent release kinetics of CK5‐GC‐NV. Sustained Gpnmb release was observed at pH 5.6, reaching a peak at 24 h, whereas only minimal release occurred at pH 7.4, thereby confirming the nanovaccine's pH‐responsive behavior (Figure 2E). This pH‐responsive disassembly facilitates the selective liberation of encapsulated cargo within the acidic microenvironment of early/late endosomes, serving as an initial prerequisite for subsequent cytosolic delivery.

**FIGURE 2:**
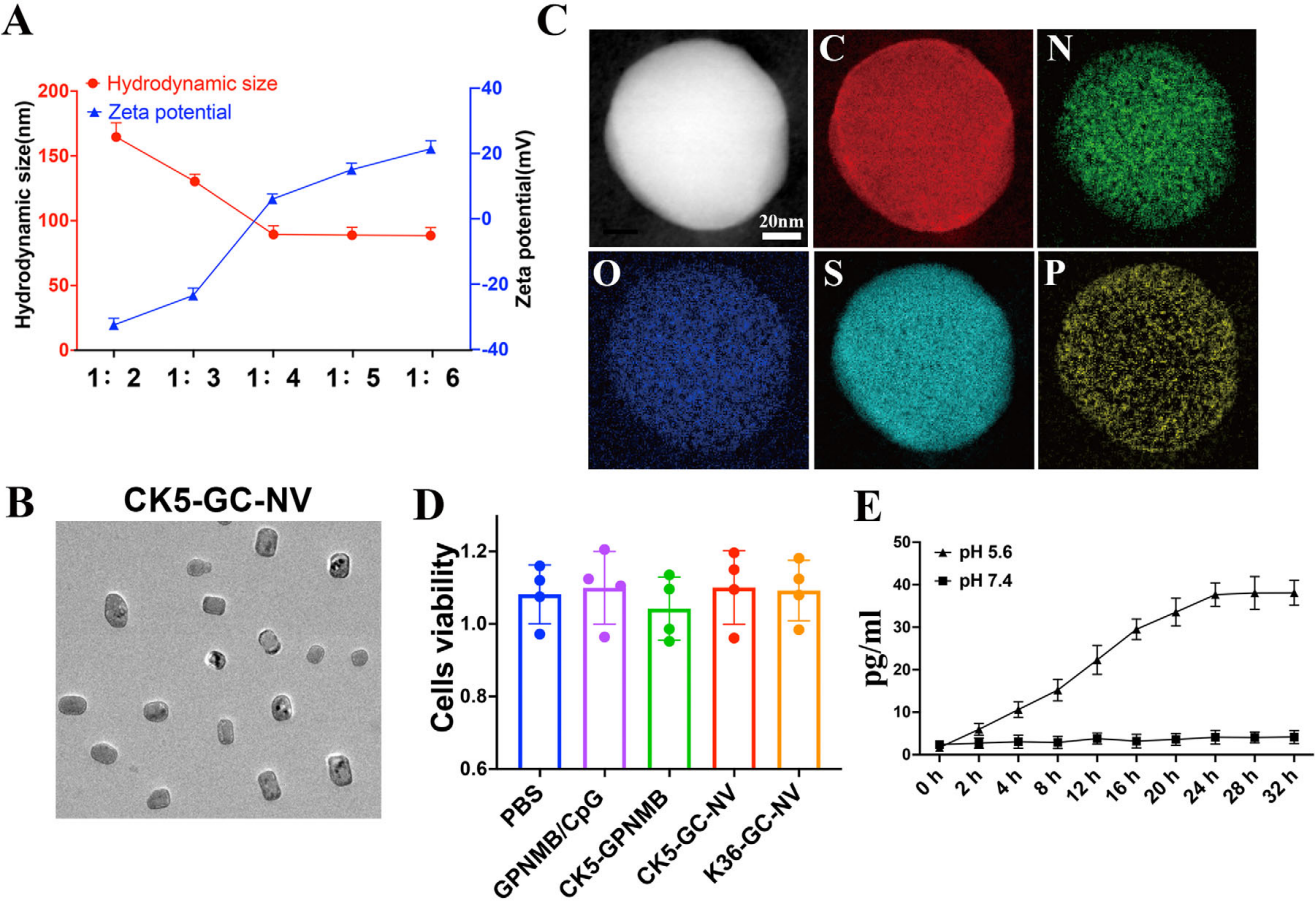
Preparation and characterization of CK5‐Gpnmb‐CpG (CK5‐GC‐NV). (A) Dynamic changes in particle size and zeta potential were observed during the assembly of nanocomplexes at varying mass ratios of CpG and CK5‐Gpnmb. (B) The CK5‐GC‐NV form nanospheres (about 90 nm) at physiological pH (pH 7.4). (C) Element mapping of CK5‐GC‐NV. (D) Cytotoxicity of PBS, Gpnmb/CpG, CK5‐Gpnmb and CK5‐GC‐NV with concentrations of 200 µg/mL was detected by CCK8 assay (*n* = 4). (E) The representative release profile of Gpnmb from CK5‐GC‐NV at different pH.

### 3.2 CK5‐GC‐NV Promotes Cytosolic Antigen Delivery and Cross‐Presentation of DCs

To assess the immunostimulatory capacity of CK5‐GC‐NV, BMDCs were incubated with various formulations, revealing significantly enhanced Gpnmb‐FITC uptake in the CK5‐GC‐NV and K36‐GC‐NV groups within 4 h (Figure S2A), attributable to the synergistic effect of the cationic protein scaffold, which alone mediated moderate internalization. Subcellular localization analysis revealed CK5‐GC‐NV's superior cytosolic delivery, evidenced by widespread, diffuse Gpnmb‐FITC fluorescence throughout the cytoplasm, whereas controls (Gpnmb/CpG, CK5‐Gpnmb, and K36‐GC‐NV) exhibited strictly punctate colocalization with LysoTracker (Figure 3A and Figure S2B). Importantly, while endosomal acidification promotes the dissociation of the electrostatic nanocomplex, actual translocation into the cytosol is mediated by the membrane‐destabilizing properties of the cationic CK5 scaffold, which facilitates endosomal membrane permeabilization to enable true endosomal escape. Once released into the cytosol, the liberated Gpnmb antigens become accessible to the cytosolic proteasomal processing machinery, providing supportive phenotypic evidence for the enhanced cell‐surface presence of H‐2K^b^–Gpnmb complexes after 48 h (Figure 3B). While this surface staining alone does not establish functional antigen cross‐presentation, the overall stimulatory and priming capacity was substantiated by downstream costimulatory marker induction and T‐cell activation assays. We subsequently assessed the maturation status of BMDCs 48 h post‐treatment. Flow cytometry revealed that CK5‐GC‐NV‐ or K36‐GC‐NV ‐treated BMDCs showed the most robust maturation, with a 3.1 ± 0.4‐fold higher CD80/CD86 expression vs. PBS/CK5‐Gpnmb and a 2.0 ± 0.3‐fold increase over Gpnmb/CpG (Figure 3C and Figure S2C). Consistent with this maturation profile, CK5‐GC‐NV or K36‐GC‐NV ‐treated BMDCs exhibited 2‐2.5‐fold enhanced IL‐12p70 secretion vs. PBS/CK5‐Gpnmb controls and a 1.5‐fold increase over Gpnmb/CpG (Figure 3D), indicative of an enhanced Th1‐promoting potential. Furthermore, CK5‐GC‐NV or K36‐GC‐NV ‐pulsed BMDCs triggered significantly enhanced proliferation of CFSE‐labeled bulk CD8^+^ T cells vs. all controls (Figure 3E and Figure S2D). Given the concurrent upregulation of CD80/86 and IL‐12, this robust proliferation reflects a superior overall T‐cell stimulatory capacity of the antigen‐bearing DCs, driven by both cognate antigen recognition (as evidenced by H‐2K^b^/Gpnmb presentation) and potent costimulatory signaling. Furthermore, TLR9 is a critical immunostimulatory pathway that is robustly activated by CpG ODN adjuvants [29] (Figure S2E). To assess TLR9 pathway activation, western blot analysis after 48 h revealed that CK5‐GC‐NV or K36‐GC‐NV treatment significantly upregulated key TLR9 pathway components (TLR9, MyD88, and p‐IRF7) vs. all controls (Figure 3F and Figure S2F), demonstrating synergistic enhancement of innate immune signaling. These baseline immunostimulatory properties—including improved uptake, endosomal escape, and TLR9 pathway activation—were observed in both CK5‐GC‐NV‐ and K36‐GC‐NV‐treated competent BMDCs, indicating that these early events represent shared delivery and adjuvant functions conferred by the cationic nanoparticle architecture and CpG complexation.

**FIGURE 3:**
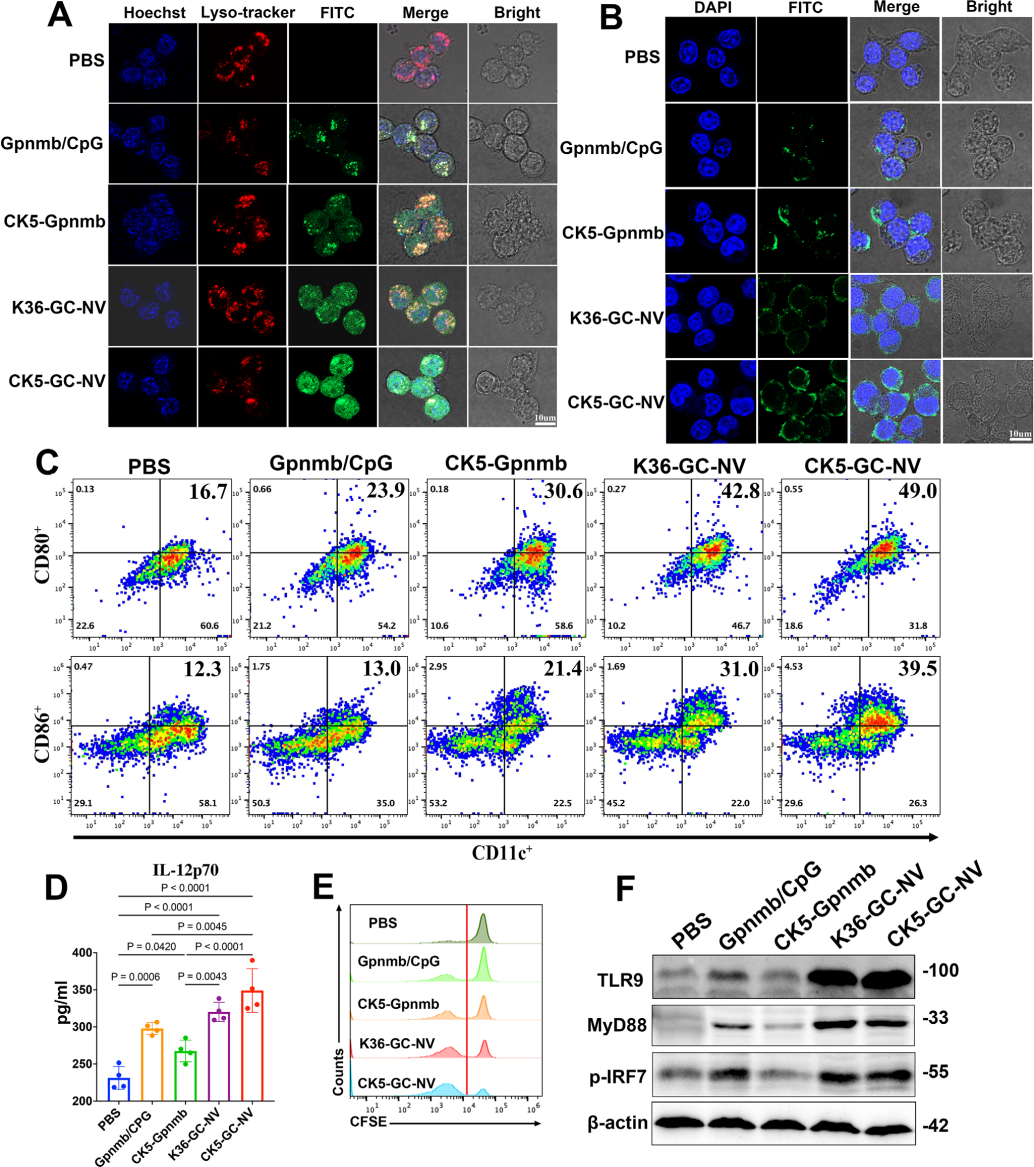
CK5‐GC‐NV elicits a robust cross‐presentation to CD8^+^ T cells. (A) The cytosolic delivery of antigen peptide induced by CK5‐GC‐NV or K36‐GC‐NV was visualized in BMDCs using confocal laser scanning microscopy (CLSM). For this analysis, BMDCs were incubated with different formulations for 24 h, and the distribution of Gpnmb‐Fluorescein Isothiocyanate (FITC) within the cells was assessed using CLSM (*n* =  4). Blue, nucleus; red, lysosomes stained with Lyso‐Tracker Deep Red; green, Gpnmb‐FITC. (B) Representative confocal images of cell‐surface Gpnmb–H‐2K^b^ complexes on intact BMDCs following a 48‐h incubation with various formulations. Blue, nucleus; green, FITC‐conjugated antibody against H‐2K^b^–Gpnmb complex (*n* =  4). (C) Representative flow cytometric analysis of CD80^+^ and CD86^+^ BMDCs gating on CD11c^+^ cells after various treatments (*n* =  4). (D) The secretion of IL‐12p70 by BMDCs following treatment with different formulations was detected by ELISA (*n* =  4). (E) The proliferation of CD8^+^ T cells was assessed by flow cytometry (*n* =  4). (F) Expression levels of key proteins (TLR9, MyD88, and p‐IRF7) in the TLR9 signaling pathway in BMDCs (*n* = 3).

### 3.3 CK5‐GC‐NV Effectively Mitigates Senescence of BMDCs

Following immunostimulation validation, we assessed CK5‐GC‐NV's senomodulatory potential via RNA‐seq analysis of CK5‐treated BMDCs (200 µg/mL, 48 h). Transcriptomic profiling identified significant downregulation of Aurka/Aurkb in CK5‐treated BMDCs (Figure 4A and Figure S3A), which was corroborated at the protein level by western blot analysis (Figure S3B), revealing a strong negative correlation between CK5 treatment and the expression of these pivotal mitotic kinases. Notably, inhibition of Aurk has been reported to downregulate the senescence‐associated PI3K/AKT signaling pathway (Figure 4B) [30, 31, 32]. We employed AlphaFold3 to predict protein–protein interactions, and the results revealed pronounced inter‐residual interactions between CK5 and the Aurka/b proteins (Figure S3C). To distinguish whether the biological activity of CK5 extends beyond generic nanoparticle‐mediated adjuvant delivery, we directly compared CK5 against its structural counterpart K36 under senescent stress. The BMDCs were treated with the cationic proteins K36 and CK5, as well as the Aurka/b inhibitor Danusertib (Danu; PHA‐739358). We evaluated their regulatory effects on the senescence‐associated PI3K/AKT signaling pathway and found that both CK5 and Danu significantly downregulated the phosphorylation levels of PI3K and AKT, whereas K36 exhibited little to modulate pathway activity (Figure S3D). Therefore, in BMDCs rendered senescent by D‐galactose (50 mm, 60 h) [15], both CK5 and Danu markedly downregulated the expression of the senescence marker proteins p16, p21, and p53 (Figure S3E). Based on these findings, senescence‐induced BMDCs were subsequently treated with different vaccine formulations, including Gpnmb/CpG, CK5–Gpnmb, K36–GC‐NV, and CK5–GC‐NV. CK5‐GC‐NV treatment significantly attenuated cellular senescence, as demonstrated by a decrease in senescence‐associated β‐galactosidase (SA‐β‐gal) activity (Figure 4C and Figure S3F) and by downregulation of p16, p21, and p53 expression (Figure 4D and Figure S3G). Intriguingly, CK5‐GC‐NV most effectively preserved the viability of D‐galactose–treated BMDCs (Figure 4E and Figure S3H). Furthermore, quantitative Western blot analysis demonstrated that total PI3K and AKT protein levels remained unchanged across experimental groups, whereas CK5‐GC‐NV treatment led to a profound reduction in both the p‐PI3K/total PI3K and p‐AKT/total AKT ratios compared with other vaccine groups (Figure 4F and Figure S3I), confirming robust suppression of pathway activation. Notably, BMDCs treated with CK5‐GC‐NV exhibited pronouncedly elevated levels of both extracellular acidification rate (ECAR) and oxygen consumption rate (OCR) compared with those in other groups (Figure 4G). CK5‐Gpnmb also exhibited moderate inhibition of the PI3K/AKT pathway, consistent with its partial anti‐senescent effects. Collectively, these findings demonstrate that CK5‐GC‐NV effectively attenuates senescence‐associated phenotypes in BMDCs, which is closely associated with the suppression of PI3K/AKT signaling, thereby preserving antigen‐presenting capacity and restoring immunocompetence compromised by aging, while exerting dual senolytic and cytoprotective effects. While our data support the involvement of the Aurka/b–PI3K/AKT axis, the precise molecular interface between CK5 and Aurka/b remains to be further elucidated through structural and biochemical approaches.

**FIGURE 4:**
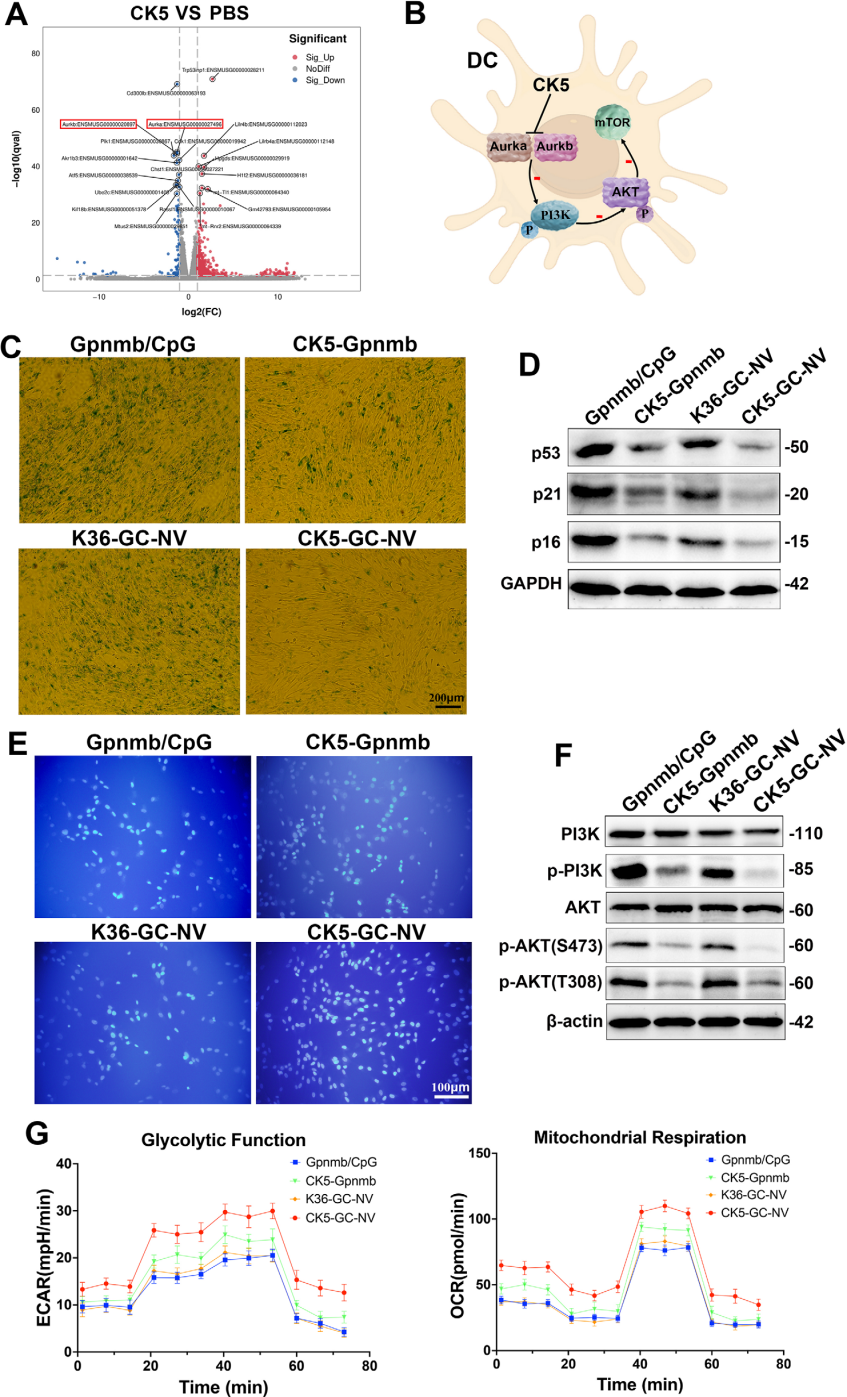
CK5‐GC‐NV alleviates aging‐related DC dysfunction via PI3K/AKT pathway suppression. (A) Volcano plot of omics analysis (RNA‐seq) comparing untreated and CK5‐treated (200 µg/mL, 48 h) BMDCs (*n* = 3). (B) Schematic illustration of Aurka/Aurkb‐mediated inhibition of the PI3K/AKT signaling pathway. (C) Representative images of senescence‐associated β‐ galactosidase (SA‐β‐gal) staining in BMDCs (*n* = 5). (D) Total protein was extracted from BMDCs treated with Gpnmb/CPG, CK5‐Gpnmb, K36‐GC‐NV, or CK5‐GC‐NV. Western blot analysis was conducted to assess the protein levels of p53, p21, and p16. GAPDH was used as an internal reference (*n* = 3). (E) BMDCs were stained with HCS NuclearMask Blue Stain to visualize and quantify viable cells (*n* = 5). (F) Western blot analysis was conducted to assess the protein levels of key components in the PI3K/AKT signaling pathway, including PI3K, p‐PI3K, AKT, and p‐AKT. β‐actin was used as an internal reference (*n* = 3). (G) Extracellular acidification rate (ECAR) and oxygen consumption rate (OCR) levels in BMDCs following treatment with different vaccine formulations (*n* = 5).

### 3.4 CK5‐GC‐NV Vaccination Exerts Potent In vivo Immunostimulatory Effects and Improves HFD‐Induced Metabolic Abnormalities

Building on the in vitro findings, in vivo imaging demonstrated the superior pharmacokinetic profile of CK5‐GC‐NV, characterized by sustained retention of Cy5‐labeled Gpnmb at the injection site for at least 120 h, in contrast to the rapid clearance (<24 h) observed with the control formulations (Gpnmb/CpG and CK5‐Gpnmb) (Figure S4A). To establish metabolic aging, C57BL/6 mice were fed HFD from 4 weeks of age and vaccinated with CK5‐GC‐NV (200 µg) or controls at 60 weeks, followed by immunological assessment at 63 weeks (Figure 5A). This prolonged HFD regimen effectively induced age‐related metabolic dysfunction prior to vaccination. Flow cytometric analysis of inguinal lymph nodes (iLNs) revealed that CK5‐GC‐NV vaccination induced a 2.9 ± 0.4‐fold increase in CD11c^+^CD80^+^ and CD11c^+^CD86^+^ mature DCs compared with all control groups (Figure 5B and Figure S4B). This in vivo DC maturation profile recapitulated the in vitro results (Figure 3C), thereby validating the potent immunostimulatory capacity of the CK5‐GC‐NV platform. To validate the in vivo mechanism, flow cytometry of the iLNs isolated from 63‐week‐old HFD‐fed mice demonstrated that CK5‐GC‐NV vaccination significantly reduced the population of p16^+^ senescent DCs (CD11c^+^ p16^+^) compared to controls (Figure S4C). Concurrently, we observed a substantial expansion of Gpnmb^+^ DCs (CD11c^+^ Gpnmb^+^) (Figure S4D). This confirms that the CK5 scaffold actively delays DC senescence to restore critical cross‐presentation and T cell activation capacities. We subsequently characterized CD8^+^ T cell activation states in the iLNs using high‐dimensional flow cytometry, given their pivotal role in cellular immunity [33, 34]. CK5‐GC‐NV vaccination markedly enhanced CD8^+^ T cell activation in iLNs, inducing 2.5–3.0‐fold increases in CD69^+^ and CD25^+^ populations compared with control groups (Figure S4E), indicative of robust early‐ and late‐stage T cell activation. Consistently, CK5‐GC‐NV vaccination resulted in a 3.8 ± 0.5‐fold increase in IFNγ^+^CD8^+^ T cells in the iLNs compared with the Gpnmb/CpG and CK5‐Gpnmb groups, and a ∼3‐fold increase relative to K36‐GC‐NV. Similarly, the frequency of GzmB^+^CD8^+^ T cells was elevated by approximately 3.1 ± 0.4‐fold, indicating the nanovaccine's capacity to elicit robust cytotoxic T lymphocyte responses and potent cellular immunity (Figure 5C and Figure S4F). To confirm the direct cytolytic capacity of vaccine‐elicited CD8^+^ T cells rather than mere Gpnmb downregulation, we performed an in vitro cytotoxicity assay. Using D‐galactose‐induced senescent 3T3‐L1 cells as surrogate targets, which we confirmed to overexpress Gpnmb (Figure S4G,H), we co‐cultured them with activated CD8^+^ T cells. Crucially, LDH release assays demonstrated that CD8^+^ T cells from CK5‐GC‐NV‐immunized mice exhibited enhanced cytotoxic activity and target‐cell lysis against Gpnmb‐expressing senescent cells, significantly outperforming control groups (Figure S4I), indicating that the enhanced killing activity was not solely driven by generalized adjuvant stimulation. Furthermore, CK5–GC–NV vaccination induced robust immunological memory, yielding 3.2 ± 0.8‐fold higher frequencies of CD8^+^CD44^+^CD62L^+^ central memory T cells (Tcm) and CD8^+^CD44^+^CD62L^−^ effector memory T cells (Tem) compared with control groups by week 70 (Figure S4J), indicating its capacity to establish durable senescence‐targeting memory T‐cell responses.

**FIGURE 5:**
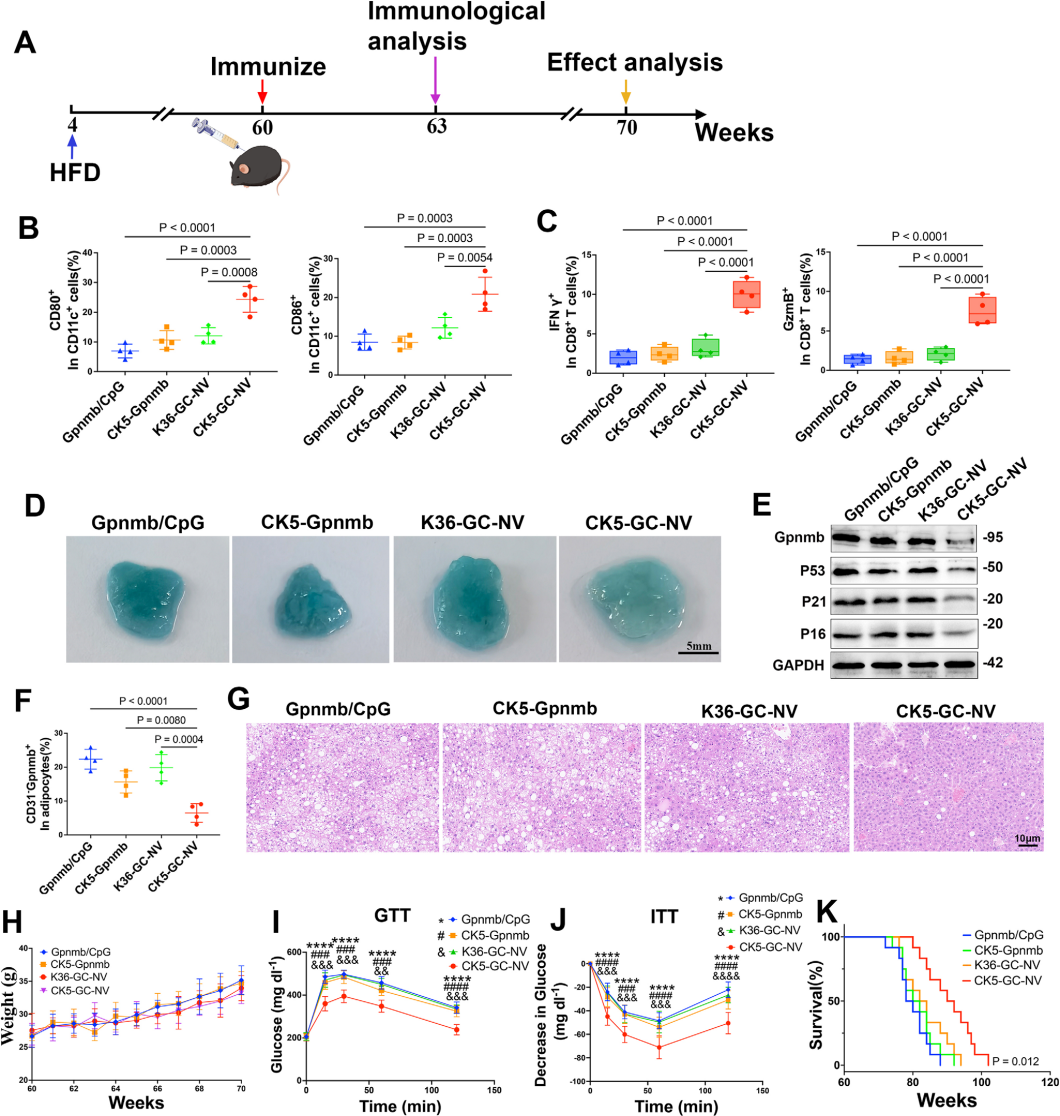
CK5‐GC‐NV exhibits robust immune responses in vivo and improves HFD‐induced metabolic abnormalities. (A) Experimental design for senescence‐targeting immunotherapy in C57BL/6 mice fed a high‐fat diet with indicated formulations of vaccines (CpG: 4nmol, CK5/K36: 8 µg, and Gpnmb: 20 µg). (B) Flow cytometric quantification data of CD11c^+^ CD80^+^ and CD11c^+^ CD86^+^ DCs in inguinal lymph nodes (iLNs) of mice (*n* = 4). (C) Flow cytometric quantification data of CD8^+^ IFNγ^+^ and CD8^+^ GzmB^+^ T lymphocytes in iLNs of mice (*n* = 4). (D) Senescence‐associated β‐galactosidase (SA‐β‐gal) staining of epididymal visceral adipose tissue (eVAT) from the indicated mouse groups (*n* = 4). (E) Representative Western blots and quantitative analysis of Gpnmb, p16, p21, and p53 expression; GAPDH was used as the loading control (*n* = 3). (F) Statistical data in CD31^−^Gpnmb^+^ adipocytes of mice (*n* = 4). (G) Representative images of hematoxylin and eosin (H&E) staining of liver tissue (*n* = 4). (H) Weekly body weight monitoring of HFD‐induced aged mice post‐immunization in the indicated groups (*n* = 6). (I) Glucose tolerance test (GTT) and (J) insulin tolerance test (ITT) performed in the indicated groups of mice (*n* = 10). For the ITT, glucose values are presented as changes relative to basal levels. (K) Kaplan–Meier survival analysis of mice maintained under high‐fat diet conditions (*n* = 10 mice per group). Median survival: CK5‐GC‐NV = 91.0 weeks (95% CI: 88.0–94.0); K36‐GC‐NV = 83.0 weeks (95% CI: 80.0–86.0); CK5‐Gpnmb = 81.0 weeks (95% CI: 78.0–84.0); Gpnmb/CpG = 79.0 weeks (95% CI: 76.0–82.0). Statistical comparison between CK5‐GC‐NV and Gpnmb/CpG: log‐rank P < 0.001, hazard ratio (HR) = 0.28 (95% CI: 0.11–0.71).

Having established the multimodal anti‐aging mechanisms of CK5‐GC‐NV, we next evaluated its therapeutic efficacy against metabolic dysfunction in high‐fat diet (HFD)‐induced aged mice (Figure 5A), with comprehensive analyses performed at week 70. CK5‐GC‐NV treatment exhibited potent senolytic activity, reducing SA‐β‐gal activity in eVAT by approximately 50% relative to other control groups (Figure 5D and Figure S5A). Meanwhile, Western blot analysis of adipose tissue lysates revealed that CK5‐GC‐NV treatment markedly downregulated the expression of key senescence markers, including p16, p21, and p53, compared with the control groups (Figure 5E and Figure S5B). CK5‐GC‐NV immunization markedly reduced the abundance of senescent cells in adipose tissue, as evidenced by an approximately 2.4 ± 0.3‐fold decrease in adipose Gpnmb protein levels (Figure 5E and Figure S5B) and a ∼3.5‐fold reduction in the CD31^−^Gpnmb^+^ cell population (Figure 5F and Figure S5C). Notably, this reduction was observed in the CD31^−^Gpnmb^+^ adipocytes rather than in endothelial cells within adipose tissue, supporting the effective reduction of Gpnmb^+^ senescent cells and the potent anti‐senescence activity of CK5‐GC‐NV. Furthermore, histomorphometric analysis demonstrated that CK5‐GC‐NV effectively ameliorated adipocyte hypertrophy, with adipocyte cross‐sectional area reduced by approximately 70% relative to the Gpnmb/CpG group and by 65% compared to the CK5‐Gpnmb and K36‐GC‐NV groups (Figure S5D). This morphological improvement was consistent with the reduced senescent cell burden, indicating an amelioration of adipose tissue dysregulation [35, 36]. Notably, H&E staining of liver sections revealed pronounced steatosis in the Gpnmb/CpG, CK5‐Gpnmb, K36‐GC‐NV groups, while hepatic steatosis was markedly attenuated in mice treated with CK5‐GC‐NV (Figure 5G). Quantitative ELISA further confirmed the systemic alleviation of senescence‐associated burden by CK5‐GC‐NV, as reflected by a 2.1 ± 0.4‐fold reduction in key circulating pro‐inflammatory SASP factors (IL‐6, TNF‐α, and IL‐1β) relative to other control groups (Figure S5E), demonstrating that the immunization did not trigger unintended chronic systemic inflammation. To comprehensively interpret the systemic metabolic improvements, we tracked the weekly body weight and food intake profiles of the HFD‐induced mice post‐immunization. Importantly, neither body weight trajectories (Figure 5H) nor average daily food intake (Figure S5F) showed significant differences among the experimental groups. This indicates that the observed metabolic benefits were not driven by caloric restriction or weight loss. Consistent with this marked improvement in adipose tissue condition, CK5‐GC‐NV treatment markedly improved core metabolic parameters compared with control groups, including enhanced glucose tolerance (Figure 5I) and reduced insulin resistance (Figure 5J). These metabolic benefits correlated with a reduction in adipocyte senescence, underscoring the functional impact of Gpnmb^+^ senescent cell clearance on aging‐associated metabolic dysfunction. To investigate the effect of CK5‐GC‐NV on survival, we assessed the lifespan of HFD‐induced mice and found that CK5‐GC‐NV significantly extended the median survival to 91 weeks, compared with 79 weeks in the Gpnmb/CpG group, 81 weeks in the CK5‐Gpnmb group, and 83 weeks in the K36‐GC‐NV group (Figure 5K).

### 3.5 CK5‐GC‐NV Immunization Ameliorates Metabolic Dysfunction in Naturally Aged Mice

To investigate the dual regulatory role of CK5‐GC‐NV in activating antigen‐specific immunity while attenuating dendritic cell senescence, mice were maintained on a standard chow diet (SCD) from 4 weeks of age until 75 weeks. The mice were then randomly assigned to four groups and immunized subcutaneously in the inguinal region with CK5‐GC‐NV or K36‐GC‐NV (Gpnmb concentration, 20µg/mL), K36‐GC‐NV+Danu (30mg/kg), or an equivalent volume of PBS. Immunological assessments were performed at 78 weeks, followed by evaluation of metabolic dysregulation at 85 weeks of age (Figure 6A). We first assessed immune activation in the lymph nodes of aged mice following immunization. Immunization with CK5‐GC‐NV or K36‐GC‐NV+Danu significantly increased the expression of the dendritic cell maturation markers CD80 and CD86 by approximately 2.8 ± 0.3‐fold compared with the K36‐GC‐NV and PBS control groups (Figure 6B and Figure S6A). In parallel, the expression of the CD8^+^ T cell activation markers CD69 and CD25 was markedly elevated (Figure S6B). Notably, IFN‐γ and GzmB expression in CD8^+^ T cells increased by at least 4.6 ± 0.5‐fold (Figure S6C), indicating robust activation of antigen‐specific, senescence‐targeting immune responses elicited by CK5‐GC‐NV and by K36‐GC‐NV+Danu in aged organisms. We next assessed the therapeutic efficacy of the intervention against age‐related metabolic dysfunction in naturally aged mice. SA‐β‐gal staining of eVAT revealed superior attenuation of senescence‐associated burden by CK5‐GC‐NV and K36‐GC‐NV+Danu, with approximately 2.7 ± 0.3‐fold lower SA‐β‐gal activity compared with PBS and K36‐GC‐NV monotherapy (Figure 6C). Moreover, CK5‐GC‐NV and K36‐GC‐NV+Danu markedly reduced the expression of senescence markers (p16, p21, and p53) as well as the senescence‐associated antigen Gpnmb in adipose tissue (Figure 6D and Figure S6D), accompanied by a significant decrease in the proportion of CD31^−^Gpnmb^+^ adipocytes (Figure 6E and Figure S6E). Furthermore, histomorphometric analysis revealed that CK5‐GC‐NV and K36‐GC‐NV+Danu exhibited superior efficacy in reducing adipocyte hypertrophy, with approximately 50%–70% decreases compared with the PBS and K36‐GC‐NV groups, respectively (Figure 6F and Figure S6F). Consistent with the alleviation of tissue senescence, these treatments also reduced circulating levels of representative SASP cytokines (IL‐6, TNF‐α, and IL‐1β) by approximately 2.6 ± 0.5‐fold compared with PBS and K36‐GC‐NV monotherapy (Figure S6G). Intriguingly, weekly metabolic profiling revealed that CK5‐GC‐NV and K36‐GC‐NV+Danu treatments did not significantly alter body weights or food consumption (Figure S6H) relative to the control groups, further confirming that the subsequent metabolic rescue is independent of feeding behavior or weight fluctuations. The CK5‐GC‐NV and K36‐GC‐NV combined with Danu conferred superior metabolic benefits, as evidenced by markedly improved glucose homeostasis (Figure 6G) and significantly reduced insulin resistance (Figure 6H) compared with PBS and K36‐GC‐NV monotherapy. While these Danusertib findings provide supportive pharmacological evidence consistent with Aurka/b involvement, systemic inhibitor administration cannot definitively isolate DC‐intrinsic actions from potential systemic off‐target effects. Therefore, the DC‐specific contribution was subsequently evaluated using an ex vivo adoptive transfer platform. Importantly, CK5‐GC‐NV significantly prolonged median survival to 113 weeks compared with control groups (K36‐GC‐NV + Danu: 111 weeks; K36‐GC‐NV: 103 weeks; PBS: 101 weeks) (Figure 6I). These survival benefits underscore the synergistic senolytic and immunomodulatory efficacy of CK5‐GC‐NV in concurrently targeting immune aging and metabolic dysfunction, highlighting its potential as a paradigm‐shifting gerotherapeutic strategy. To further evaluate the systemic safety and potential off‐target toxicity of the senolytic nanovaccine, histopathological assessments of major vital organs were performed. Systematic H&E histological examination of major organs (heart, liver, spleen, lung, and kidney) from naturally aged mice revealed preserved tissue architecture without observable gross morphological damage or overt inflammatory lesions following CK5‐GC‐NV vaccination (Figure S6I). While these preliminary histological profiles suggest a favorable morphological tolerance, further comprehensive evaluations will be necessary to rule out subclinical toxicities.

**FIGURE 6:**
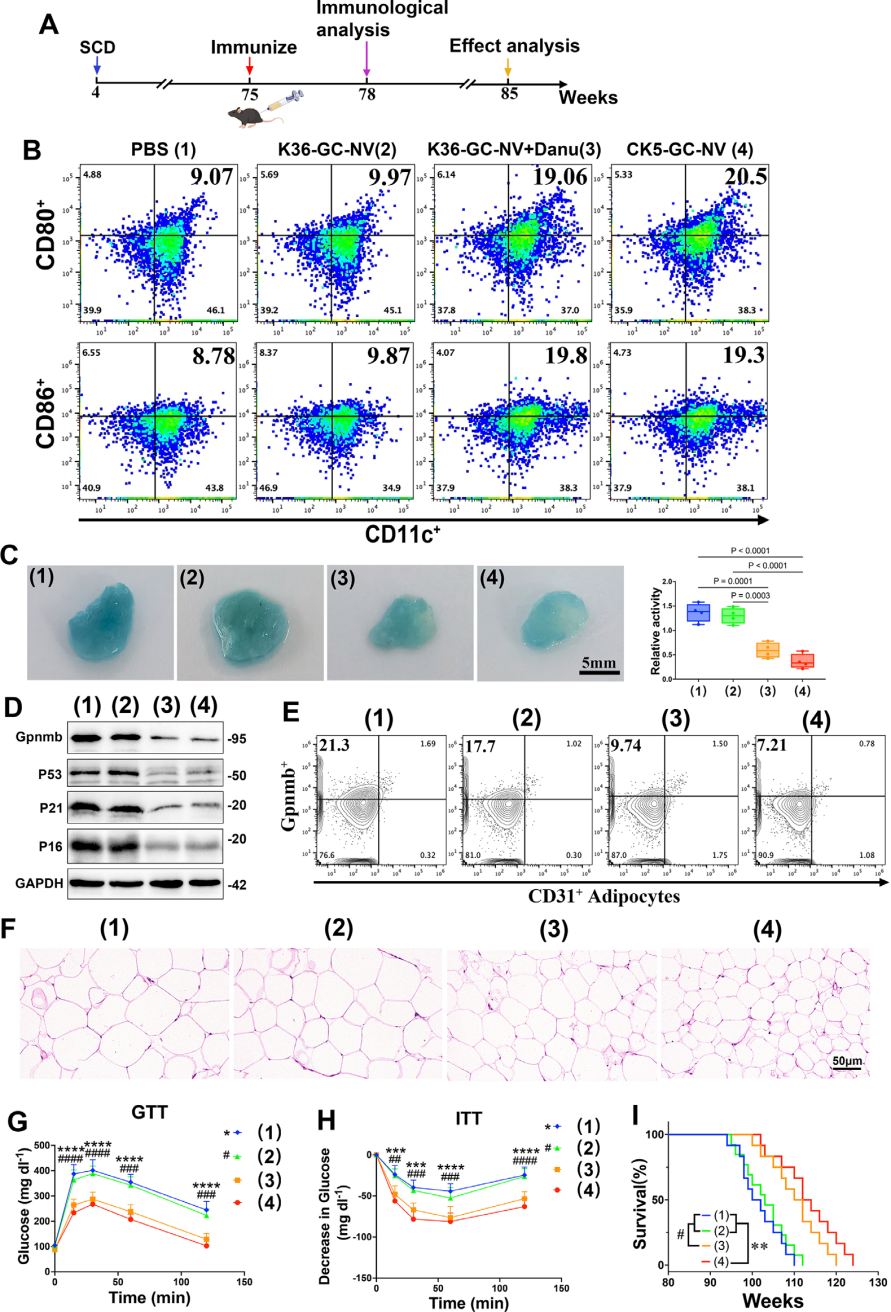
The CK5‐GC‐NV significantly attenuated adipose tissue senescence and aging‐related metabolic abnormalities in naturally aged mice. (A) Schematic overview of the subcutaneous immunization and efficacy evaluation protocol in mice fed an SCD, with defined vaccine formulations (CpG, 4 nmol; CK5/K36, 8 µg; Gpnmb, 20 µg; Danu, 30 mg/kg). (B) Representative flow cytometric analysis of CD11c^+^ CD80^+^ and CD11c^+^ CD86^+^ DCs in LNs of mice (*n* = 4). (C) Representative SA‐β‐gal staining of epididymal visceral adipose tissue (eVAT) from different groups and corresponding quantification of SA‐β‐gal activity (*n* = 4). (D) Western blot analysis was performed to evaluate the activation levels of Gpnmb, p16, p21, and p53, utilizing specific antibodies for each target protein (*n* = 3). GAPDH served as an internal reference for normalization. (E) Adipose tissue was processed into single‐cell suspensions, and flow cytometry was employed to analyze CD31^−^Gpnmb^+^ adipocytes (*n* = 4). (F) Representative images of hematoxylin and eosin (H&E) staining of visceral adipose tissue (>150 adipocytes per animal) (*n* =  10). Glucose tolerance test (GTT) (G) and insulin tolerance test (ITT) (H) were conducted in different groups of mice (*n* =  10), with ITT data expressed as changes from basal glucose levels. (I) Kaplan–Meier survival analysis of naturally aged mice (*n* = 10 mice per group). Median survival: CK5‐GC‐NV = 113.0 weeks (95% CI: 110.0–116.0); K36‐GC‐NV+Danu = 111.0 weeks (95% CI: 108.0–114.0); K36‐GC‐NV = 103.0 weeks (95% CI: 100.0–106.0); PBS = 101.0 weeks (95% CI: 98.0–104.0). Statistical comparison between CK5‐GC‐NV and PBS: log‐rank P < 0.001, HR = 0.24 (95% CI: 0.09–0.63).

### 3.6 The K36‐GC‐NV–Pulsed DCs Vaccine Demonstrated Therapeutic Efficacy in Ameliorating Metabolic and Senescent Phenotypes Comparable to That of CK5‐GC‐NV Vaccination

To determine whether CK5‐GC‐NV enhances antigen‐specific cellular immunity against senescent markers via DC modulation in aged mice, BMDCs were loaded with K36‐GC‐NV at a dose equivalent to CK5‐GC‐NV to generate a DC vaccine (K36‐GC‐NV–DC), which was administered by inguinal subcutaneous injection in a D‐galactose–induced aging model (Figure 7A). We found that immunization with either CK5‐GC‐NV or K36‐GC‐NV–DC significantly reduced SA‐β‐gal activity in eVAT compared with antigen‐unloaded DCs (Ctl‐DC) (Figure 7B). Moreover, both CK5‐GC‐NV and K36‐GC‐NV–DC markedly downregulated the expression of senescence markers (p16, p21, and p53) and the senescence‐associated antigen Gpnmb in adipose tissue (Figure 7C and Figure S7A), accompanied by a significant reduction in the proportion of CD31^−^Gpnmb^+^ adipocytes (Figure 7D and Figure S7B). Concordantly, histomorphometric analysis revealed a substantial attenuation of adipocyte hypertrophy, with reductions of approximately 50%–60% compared with the Ctl‐DC group (Figure 7E). Furthermore, both CK5‐GC‐NV and K36‐GC‐NV–DC exhibited superior metabolic efficacy, demonstrated by enhanced glucose homeostasis (Figure 7F) and attenuated insulin resistance (Figure 7G) relative to Ctl‐DC. Notably, CK5‐GC‐NV immunization resulted in a significant extension of median survival to 112 weeks, exceeding that observed in the K36‐GC‐NV–DC (109 weeks) and Ctl‐DC (103.5 weeks) groups (Figure 7H). Importantly, unlike systemic Danusertib administration, this ex vivo DC vaccination paradigm directly isolates the contribution of dendritic cells from systemic bystander effects. The finding that adoptively transferred K36‐GC‐NV–pulsed DCs recapitulated the therapeutic efficacy of CK5‐GC‐NV provides direct functional evidence that modulating DC‐intrinsic competence is sufficient to orchestrate the downstream clearance of senescent cells and ameliorate systemic metabolic decline.

**FIGURE 7:**
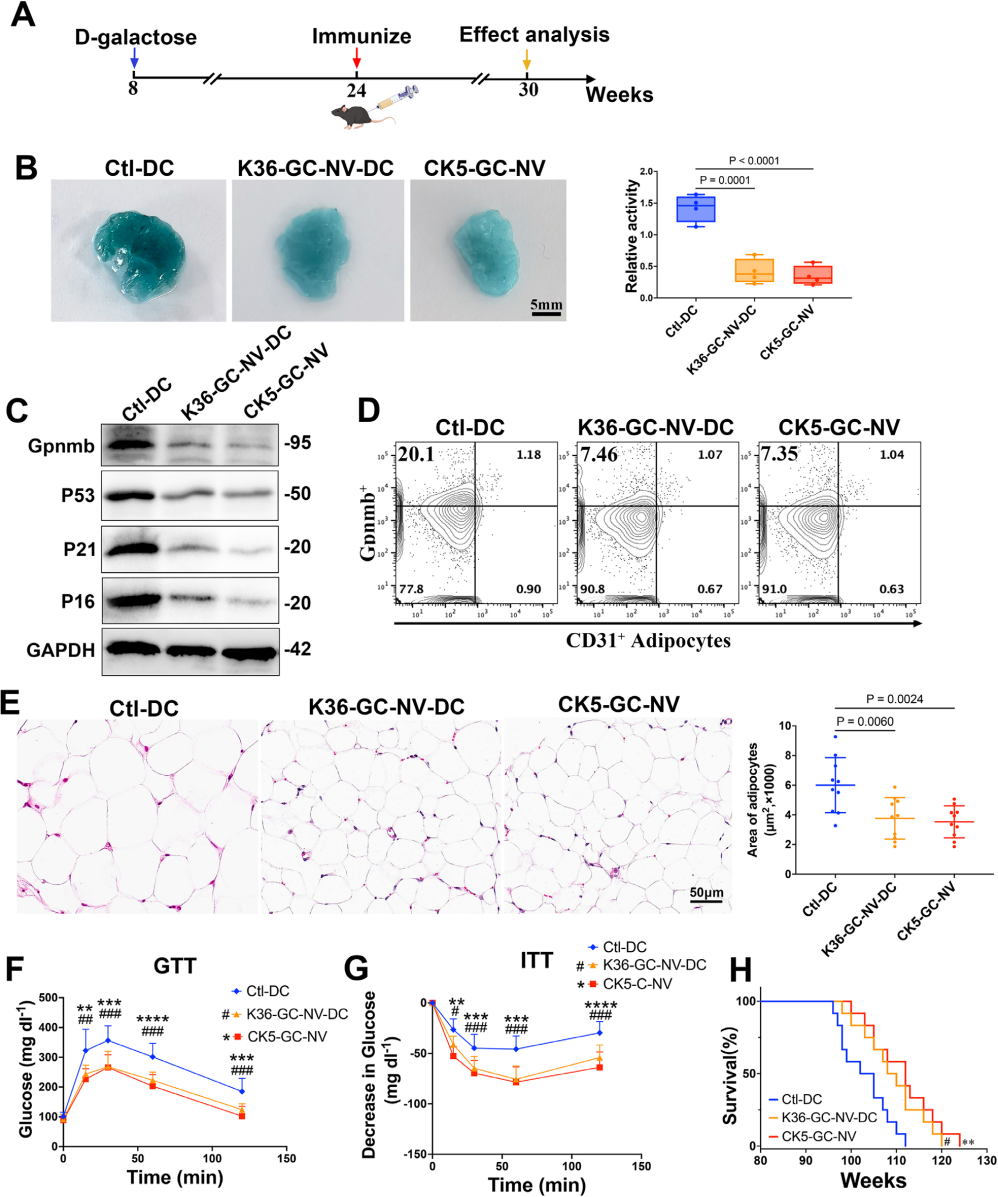
K36‐GC‐NV–DC or CK5‐GC‐NV immunization effectively mitigated aging‐related metabolic dysfunction in a D‐galactose–induced aging model. (A) Schematic diagram of the experimental timeline for subcutaneous immunization and efficacy assessment in a D‐galactose‐induced aging mouse model. (B) Representative SA‐β‐gal staining images of eVAT across experimental groups, with corresponding quantification of SA‐β‐gal activity (*n* = 4). (C) Representative Western blots and densitometric quantification of Gpnmb, p16, p21, and p53 protein levels, normalized to GAPDH (*n* = 3). (D) Adipose tissue was enzymatically digested into single‐cell suspensions, and CD31^−^ Gpnmb^+^ adipocytes were quantified by flow cytometry (*n* = 4). (E) Representative H&E‐stained sections of visceral adipose tissue, with quantification of adipocyte cross‐sectional area based on >150 adipocytes per animal (*n* = 10). Glucose tolerance test (GTT) (F) and insulin tolerance test (ITT) (G) analyses in mice from different experimental groups (*n* = 10), with ITT results expressed as changes from baseline glucose levels. (H) Kaplan–Meier survival analysis of mice in the D‐galactose–induced model (*n* = 10 mice per group). Median survival: CK5‐GC‐NV = 112.0 weeks (95% CI: 109.0–115.0); K36‐GC‐NV–DC = 109.0 weeks (95% CI: 106.0–112.0); Ctl‐DC = 103.5 weeks (95% CI: 100.0–107.0). Statistical comparison between CK5‐GC‐NV and Ctl‐DC: log‐rank P < 0.01, HR = 0.31 (95% CI: 0.12–0.79).

## 4 Discussion

Cellular senescence is increasingly recognized as a central driver of organismal aging and age‐associated metabolic dysfunction [11, 37]. The senolytic vaccination has emerged as a promising strategy for the selective elimination of senescent cells [7, 8, 38]. However, the translation of senolytic vaccines is fundamentally bottlenecked by the inherent poor immunogenicity of senescence‐associated antigens and the systemic functional decline of host DCs in aged microenvironments [39, 40]. In this study, we developed a multifunctional bi‐adjuvant nanovaccine, CK5‐GC‐NV, that integrates antigen delivery, innate immune stimulation, and the functional rescue of aged DCs within a single nanoengineered platform. We demonstrated that the specific structural and physicochemical properties of the CK5 protein scaffold inherently reprogram DC senescence, thereby establishing a highly potent immunosenolytic platform.

A key conceptual advance of this work lies in the recognition that effective senolytic vaccination in aging cannot rely solely on improving antigen immunogenicity. Previous studies have shown that senescence‐associated antigens such as Gpnmb or CD153 can serve as actionable immune targets for the selective elimination of senescent cells [7, 8, 41]. However, in aged hosts, antigen‐specific immune responses are intrinsically limited by immunosenescence, particularly by the impaired priming capacity of DCs [21, 40, 42]. Our data suggest that this is not merely an accompanying phenomenon, but rather a central bottleneck that determines the therapeutic ceiling of senolytic vaccines. By directly targeting DC dysfunction, CK5‐GC‐NV overcomes this bottleneck and converts a conventionally weak senescence‐associated antigen into an effective trigger of cytotoxic immunity. Mechanistically, our study reveals that CK5 possesses intrinsic senomodulatory activity beyond its role as a structural carrier. Accumulating evidence indicates that senescent DCs exhibit cell‐intrinsic hyperactivation of the PI3K/AKT pathway, which paralyzes their capacity to prime CD8^+^ T‐cell responses against seno‐antigens [43, 44]. Remarkably, our transcriptomic and proteomic analyses revealed that CK5 treatment is robustly associated with the downregulation of the mitotic kinases Aurka/b. Given that Aurka/b inhibition has been linked to the suppression of the senescence‐associated PI3K/AKT signaling cascade, the internalization of CK5‐GC‐NV effectively is correlated with dampened p‐PI3K and p‐AKT levels, representing a proposed rather than definitively established linear pathway. This material‐induced signaling blockade effectively attenuates senescence‐associated phenotypes in DCs, restoring cellular metabolism (ECAR and OCR) and rescuing their antigen‐presenting competence. Nanoparticle‐facilitated antigen uptake, endosomal release, and TLR9 activation are general features shared by both CK5‐ and K36‐based nanocomplexes. The indispensability of this specific material‐biology interaction was unequivocally validated by substituting CK5 with the structurally distinct K36 cationic protein. The K36‐GC‐NV counterpart failed to mitigate DC senescence and required co‐formulation with a pharmacological Aurka/b inhibitor (Danusertib) to match the immunomodulatory and senolytic efficacy of CK5‐GC‐NV. Although systemic Danusertib administration provides supportive pharmacological evidence linking Aurka/b inhibition to pathway suppression, the definitive isolation of DC‐specific functionality was substantiated by our ex vivo DC adoptive transfer experiments (Figure 7), which bypassed systemic confounding factors to confirm that functional DC priming drives the systemic anti‐senescence response.

The restoration of DC function directly translated into profound systemic physiological benefits. The functional consequence of this restoration of DC immunocompetence is the elicitation of a potent cytotoxic T‐lymphocyte (CTL) response directed against Gpnmb‐expressing senescent cells [12, 45]. Importantly, the long‐term senolytic efficacy of a single CK5‐GC‐NV vaccination is not dependent on the sustained survival of the antigen‐presenting DCs, which naturally possess a short lifespan. Instead, the DCs with rescued antigen‐presenting function effectively prime CD8^+^ T cells, culminating in the generation of a durable pool of Gpnmb‐specific central (Tcm) and effector (Tem) memory T cells. This long‐lived memory T cell population provides continuous immunosurveillance, enabling the persistent clearance of newly generated senescent cells. The subsequent reduction in senescent cell burden and circulating senescence‐associated secretory phenotype (SASP) factors fundamentally reshaped the aging metabolic microenvironment. This was functionally manifested by the rescue of high‐fat diet‐induced and age‐associated metabolic abnormalities, yielding marked improvements in glucose tolerance and insulin sensitivity, alongside a reduction in adipocyte hypertrophy and hepatic steatosis. Furthermore, the synergistic senolytic and immunomodulatory efficacy of CK5‐GC‐NV culminated in a significant extension of median lifespan in both metabolic and natural aging models. Importantly, our platform bifurcates senescent cell fates through distinct mechanisms: intracellular CK5 provides senomorphic protection to maintain DC immunocompetence, while vaccine‐induced CD8^+^ CTLs extrinsically clear Gpnmb^+^ senescent adipocytes. Reflecting the multifaceted nature of senescence, this strategy decouples cytoprotective senomorphic modulation in immune effectors from cytotoxic senolysis in parenchymal tissues, offering a refined framework for multimodal senotherapies.

Despite these advances, several limitations should be noted. First, because Gpnmb is basally expressed in normal myeloid compartments, the long‐term safety of persistent self‐reactive memory T cells and potential on‐target/off‐senescent toxicities warrant longitudinal surveillance. Second, while our transcriptomic and pharmacological data associate CK5 with Aurka/b and PI3K/AKT downregulation, biophysical target‐engagement assays (e.g., SPR or co‐IP) and genetic epistasis remain essential to establish formal molecular causality. Third, chemically induced D‐galactose BMDC stress does not fully recapitulate physiological aging; future studies should validate this signaling cascade in naturally aged DCs alongside broader markers like γ‐H2AX and Lamin B1. In addition, although ex vivo DC adoptive transfer (Figure 7) and contact‐dependent cytotoxicity assays functionally support the DC–CD8^+^ T cell axis, future studies employing genetic knockout (e.g., TLR9‐ or Gpnmb‐deficient) and CD8^+^ T‐cell depletion models are needed to definitively substantiate this axis in vivo. Finally, our evidence for senolysis relies on static surrogate readouts and Gpnmb^+^ cell reduction rather than dynamic tracking of bona fide senescent targets. Validating these findings using genetic ablation models (e.g., INK‐ATTAC or p21‐ATTAC) represents a critical future step to confirm targeted senolysis while sparing physiological Gpnmb‐expressing lineages.

## 5 Conclusions

Collectively, our work presents a paradigm‐shifting bi‐adjuvant nanovaccine that bridges supramolecular biomaterials with geroscience. By rationally designing a self‐assembling nanoscaffold that not only co‐delivers antigens and adjuvants but inherently acts as an Aurka/b‐targeted senomodulator, we effectively ameliorated age‐associated DC dysfunction and orchestrated a potent senolytic immune response. This active biomaterial strategy provides a versatile and highly efficient framework for the future development of precision senolytic therapeutics against aging‐related metabolic diseases.

## Author Contributions

**Qiliang Yin**, **Qingxu Song**, **Xiaoxue Du**, and **Wenbo Dong** were involved in the study design and wrote the manuscript. **Qingxu Song**, **Xiaoxue Du**, **Wenbo Dong**, **Ying Wang**, and **Xiaojing Wang** performed the experiments and analyzed the data. **Qiliang Yin** and **Xiaojing Wang** contributed to the review of the manuscript. All authors were involved in revising the article critically and gave approval for the final manuscript to be published.

## Ethics Statement

All animal experiments were conducted in accordance with the ARRIVE guidelines and approved by the Animal Ethics Committee of the First Hospital of Jilin University (Approval Number: 20260225‐121).

## Conflicts of Interest

The authors declare no conflicts of interest.

## Supporting information




**Supporting File**: advs78545‐sup‐0001‐SuppMat.docx.

## Data Availability

The data that support the findings of this study are available from the corresponding author upon reasonable request.
