# Supplementary material for: A Bi‐Adjuvant Senolytic Nanovaccine Modulating Dendritic Cell Senescence Ameliorates Aging‐Related Metabolic Dysfunction
Source: Adv Sci (Weinh). 2026 Oct 28:e78545. Online ahead of print. doi: 10.1002/advs.78545 (PMC13715673; doi:10.1002/advs.78545)
Supplement: Supplementary file 1 [file ADVS-9999-e78545-s001.docx]

**Supplementary materials**

MATERIALS AND METHODS

**Table S1.** Gene sequences

| Gene name | Sequences |
| --- | --- |
| Gpnmb | aggaggggagacggcaggtggaaggac |
| CK5 | atggctagcggctcttctgcggctgcggcggcagcggcggcatctggtccgggtggttatggtccggaaaaccagggtccgtctggtccgggtggctatggtccgggcggtccagtaccgggtaaaggcgtgccgggcaaaggtgttccaggcaaaggcgttccgggcaaaggcgtgccgggtaaaggcactagcggctcttctgcggctgcggcggcagcggcggcatctggtccgggtggttatggtccggaaaaccagggtccgtctggtccgggtggctatggtccgggcggtccagtaccgggtaaaggcgtgccgggcaaaggtgttccaggcaaaggcgttccgggcaaaggcgtgccgggtaaaggcactagcggctcttctgcggctgcggcggcagcggcggcatctggtccgggtggttatggtccggaaaaccagggtccgtctggtccgggtggctatggtccgggcggtccagtaccgggtaaaggcgtgccgggcaaaggtgttccaggcaaaggcgttccgggcaaaggcgtgccgggtaaaggcactagcggctcttctgcggctgcggcggcagcggcggcatctggtccgggtggttatggtccggaaaaccagggtccgtctggtccgggtggctatggtccgggcggtccagtaccgggtaaaggcgtgccgggcaaaggtgttccaggcaaaggcgttccgggcaaaggcgtgccgggtaaaggcactagcggctcttctgcggctgcggcggcagcggcggcatctggtccgggtggttatggtccggaaaaccagggtccgtctggtccgggtggctatggtccgggcggtccagtaccgggtaaaggcgtgccgggcaaaggtgttccaggcaaaggcgttccgggcaaaggcgtgccgggtaaaggcactagcggctcttctgcggctgcggcggcagcggcggcatctggtccgggtggttatggtccggaaaaccagggtccgtctggtccgggtggctatggtccgggcggtccagtaccgggtaaaggcgtgccgggcaaaggtgttccaggcaaaggcgttccgggcaaaggcgtgccgggtaaaggcactagccaccatcaccatcatcattga |
| CK5-Gpnmb | atgaggaggggagacggcaggtggaaggacggcagcggcaggaggggagacggcaggtggaaggacggcgcggggccgggcgtgggtatggctagcggctcttctgcggctgcggcggcagcggcggcatctggtccgggtggttatggtccggaaaaccagggtccgtctggtccgggtggctatggtccgggcggtccagtaccgggtaaaggcgtgccgggcaaaggtgttccaggcaaaggcgttccgggcaaaggcgtgccgggtaaaggcactagcggctcttctgcggctgcggcggcagcggcggcatctggtccgggtggttatggtccggaaaaccagggtccgtctggtccgggtggctatggtccgggcggtccagtaccgggtaaaggcgtgccgggcaaaggtgttccaggcaaaggcgttccgggcaaaggcgtgccgggtaaaggcactagcggctcttctgcggctgcggcggcagcggcggcatctggtccgggtggttatggtccggaaaaccagggtccgtctggtccgggtggctatggtccgggcggtccagtaccgggtaaaggcgtgccgggcaaaggtgttccaggcaaaggcgttccgggcaaaggcgtgccgggtaaaggcactagcggctcttctgcggctgcggcggcagcggcggcatctggtccgggtggttatggtccggaaaaccagggtccgtctggtccgggtggctatggtccgggcggtccagtaccgggtaaaggcgtgccgggcaaaggtgttccaggcaaaggcgttccgggcaaaggcgtgccgggtaaaggcactagcggctcttctgcggctgcggcggcagcggcggcatctggtccgggtggttatggtccggaaaaccagggtccgtctggtccgggtggctatggtccgggcggtccagtaccgggtaaaggcgtgccgggcaaaggtgttccaggcaaaggcgttccgggcaaaggcgtgccgggtaaaggcactagcggctcttctgcggctgcggcggcagcggcggcatctggtccgggtggttatggtccggaaaaccagggtccgtctggtccgggtggctatggtccgggcggtccagtaccgggtaaaggcgtgccgggcaaaggtgttccaggcaaaggcgttccgggcaaaggcgtgccgggtaaaggcactagccaccatcaccatcatcattga |
| CpG ODN 1826 | tccatgacgttcctgacgtt |

**Table S2.** Sequences of proteins and peptides

| Protein name | Sequences |
| --- | --- |
| Gpnmb | RRGDGRWKD |
| CK5 | MASGSSAAAAAAAASGPGGYGPENQGPSGPGGYGPGGPVPGKGVPGKGVPGKGVPGKGVPGKGTSGSSAAAAAAAASGPGGYGPENQGPSGPGGYGPGGPVPGKGVPGKGVPGKGVPGKGVPGKGTSGSSAAAAAAAASGPGGYGPENQGPSGPGGYGPGGPVPGKGVPGKGVPGKGVPGKGVPGKGTSGSSAAAAAAAASGPGGYGPENQGPSGPGGYGPGGPVPGKGVPGKGVPGKGVPGKGVPGKGTSGSSAAAAAAAASGPGGYGPENQGPSGPGGYGPGGPVPGKGVPGKGVPGKGVPGKGVPGKGTSGSSAAAAAAAASGPGGYGPENQGPSGPGGYGPGGPVPGKGVPGKGVPGKGVPGKGVPGKGTSHHHHHH |
| CK5-Gpnmb | MRRGDGRWKDGSGRRGDGRWKDGAGPGVGMASGSSAAAAAAAASGPGGYGPENQGPSGPGGYGPGGPVPGKGVPGKGVPGKGVPGKGVPGKGTSGSSAAAAAAAASGPGGYGPENQGPSGPGGYGPGGPVPGKGVPGKGVPGKGVPGKGVPGKGTSGSSAAAAAAAASGPGGYGPENQGPSGPGGYGPGGPVPGKGVPGKGVPGKGVPGKGVPGKGTSGSSAAAAAAAASGPGGYGPENQGPSGPGGYGPGGPVPGKGVPGKGVPGKGVPGKGVPGKGTSGSSAAAAAAAASGPGGYGPENQGPSGPGGYGPGGPVPGKGVPGKGVPGKGVPGKGVPGKGTSGSSAAAAAAAASGPGGYGPENQGPSGPGGYGPGGPVPGKGVPGKGVPGKGVPGKGVPGKGTSHHHHHH |

**Table S3**. Comprehensive list of antibodies and reagents

| **Target Antigen** | **Application** | **Host/ Clonality** | **Clone** | **Vendor** | **Catalog No.** | **Dilution** |
| --- | --- | --- | --- | --- | --- | --- |
| CD11c | Flow Cytometry | Hamster mAb | N418 | BioLegend | 117310 | 1:100 |
| CD80 | Flow Cytometry | Hamster mAb | 16-10A1 | BioLegend | 104706 | 1:200 |
| CD86 | Flow Cytometry | Rat mAb | GL-1 | BioLegend | 105008 | 1:200 |
| CD8a | Flow Cytometry | Rat mAb | 53-6.7 | BioLegend | 100706 | 1:200 |
| CD69 | Flow Cytometry | Hamster mAb | H1.2F3 | BioLegend | 104508 | 1:100 |
| CD25 | Flow Cytometry | Rat mAb | PC61 | BioLegend | 102012 | 1:100 |
| IFN-γ | Flow Cytometry | Rat mAb | XMG1.2 | BioLegend | 505808 | 1:100 |
| Granzyme B | Flow Cytometry | Mouse mAb | QA16A02 | BioLegend | 372204 | 1:100 |
| CD44 | Flow Cytometry | Rat mAb | IM7 | BioLegend | 103028 | 1:200 |
| CD62L | Flow Cytometry | Rat mAb | MEL-14 | BioLegend | 104412 | 1:200 |
| CD31 | Flow Cytometry | Rat mAb | 390 | BioLegend | 102408 | 1:200 |
| p16 | Flow Cytometry | Mouse mAb | 2D9A12 | BD Biosciences | 565495 | 1:100 |
| Gpnmb | Flow Cytometry | Rat mAb | CTSRE-1 | Thermo Fisher | 50-5708-82 | 1:100 |
| H-2Kᵇ–Gpnmb | CLSM | Mouse mAb | Polyclonal | GENEWIZ | Custom | 1:200 |
| Gpnmb | Western Blot | Rabbit Poly | Polyclonal | Proteintech | 11986-1-AP | 1:1000 |
| p53 | Western Blot | Mouse mAb | 1C12 | Proteintech | 60283-2-Ig | 1:1000 |
| p21 | Western Blot | Rabbit Poly | Polyclonal | Proteintech | 28248-1-AP | 1:1000 |
| p16 | Western Blot | Rabbit Poly | Polyclonal | Proteintech | 10883-1-AP | 1:1000 |
| GAPDH | Western Blot | Mouse mAb | 1E6D9 | Proteintech | 60004-1-Ig | 1:5000 |
| β-actin | Western Blot | Mouse mAb | 2D4H5 | Proteintech | 66009-1-Ig | 1:5000 |
| Aurka | Western Blot | Mouse mAb | 1F8 | Abcam | ab1287 | 1:1000 |
| Aurkb | Western Blot | Rabbit Poly | Polyclonal | Abcam | ab2254 | 1:1000 |
| p-AKT (S473) | Western Blot | Rabbit mAb | EP2109Y | Abcam | ab81283 | 1:1000 |
| p-AKT (T308) | Western Blot | Rabbit mAb | 244F9 | Abcam | ab38449 | 1:1000 |
| Total AKT | Western Blot | Rabbit Poly | Polyclonal | Abcam | ab179463 | 1:1000 |
| Total PI3K | Western Blot | Rabbit Poly | Polyclonal | Abcam | ab278545 | 1:1000 |
| **Target Antigen/Complex** | **Host / Clonality** | **Conjugate** | **Catalog / Lot No.** | **Source / Provider** | **Working Dilution** | **QC Parameters** |
| H-2Kᵇ–Gpnmb complex (peptide: RRGDGRWKD | Rabbit Polyclonal (Affinity Purified) | FITC | Cat# GW-MHC0826  Lot# GW20240315-QC02 | GENEWIZ (Azenta Life Sciences, Suzhou, China) | IF: 1:100 (5–10 μg/mL) | QC ELISA: Titer > 1:64,000 against cognate H-2Kᵇ–Gpnmb monomer.  Counter-screening Specificity: Signal ratio > 25:1 against irrelevant control monomer (H-2Kᵇ–OVA₂₅₇₋₂₆₄, SIINFEKL) and empty H-2Kᵇ heavy chain.  Purity: > 95% by SDS-PAGE. FITC/Protein (F/P) molar ratio: 3.2–4.0. |

**Figure S1.** **(A)** Schematic diagram of the CK5-Gpnmb gene construction. **(B)** Exploring the appropriate proportions of CpG and CK5-Gpnmb to form optimal nanocomplexes with suitable CpG packing efficiency. **(C)** Energy dispersive spectroscopy (EDS) was utilized to analyze the primary elemental composition of CK5-GC-NV. **(D)** The K36-GC-NV form nanospheres (about 80 nm) at physiological pH (pH 7.4). **(E)** Cytotoxicity of CK5-GC-NV with different concentrations (0-300 μg/mL) was detected by CCK8 assay (n = 4).

**Figure S2.** **(A)** BMDCs were incubated with different formulations for 4 hours, and the uptake of Gpnmb-FITC in the BMDCs was observed using CLSM (n=4). Blue, nucleus; green, Gpnmb-FITC. **(B)** The quantification of fluorescence co-localization between Gpnmb-FITC and LysoTracker (n=4). **(C)** Quantitative analysis of CD80 and CD86 expression on CD11c^+^BMDCs (n=4). **(D)** Quantitative data on proliferation of CD8^+^ T cells (n=4). **(E)** Schematic illustration of CpG ODN–mediated activation of the TLR9 signaling pathway. **(F)** Western blot analysis was performed to evaluate the protein levels of key components in the TLR9 signaling pathway, including TLR9, MyD88, and phosphorylated IRF7 (p-IRF7). β-Actin served as an internal control, and the protein levels of TLR9, MyD88, and p-IRF7 were quantified (n = 3). The data are expressed as mean ± SD, using GraphPad software for statistical analysis, and the differences were assessed using one-way ANOVA: **p* <0.05, ***p* <0.01, ****p* <0.001 and *****p* < 0.0001.

**Figure S3. (A)** Heatmap of omics analysis (RNA-seq) comparing untreated and CK5-treated (200 μg/mL, 48 h) BMDCs (n=3). **(B)** Total protein was extracted from BMDCs treated with PBS, K36 or CK5, followed by Western blot analysis to assess the expression levels of Aurka and Aurkb using specific antibodies. β-Actin served as an internal control, and the protein levels of Aurka and Aurkb were quantified (n = 3). **(C)** Prediction of CK5–Aurka/Aurkb protein interactions using AlphaFold3. **(D)** Total protein was extracted from BMDCs treated with PBS, K36, CK5, and Danu, followed by Western blot analysis to determine the expression of PI3K, p-PI3K, AKT, and p-AKT using specific antibodies. β-Actin was used as a loading control, and protein levels were quantified (n = 3). **(E)** Western blot analysis was conducted to assess the protein levels of p53, p21 and p16. GAPDH was used as an internal reference, and protein levels were quantified (n=3). **(F)** Quantitative analysis of senescence-associated β-galactosidase (SA-β-gal) staining in BMDCs (n = 5). **(G)** The quantification of protein levels from western blot analysis of p53, p21 and p16 (n= 3). **(H)** BMDC viability was assessed using HCS NuclearMask™ Blue staining, with quantitative analysis of viable cell numbers (n= 5). **(I)** Densitometric quantification of the ratios of phosphorylated PI3K to total PI3K (p-PI3K/total PI3K) and phosphorylated AKT to total AKT (p-AKT/total AKT) across the indicated treatment groups. Total PI3K and AKT levels remained unaltered across all conditions (n = 3). All data are expressed as mean ± SD, using GraphPad software for statistical analysis, and the differences were assessed using one-way ANOVA: **p* <0.05, ***p* <0.01, ****p* <0.001 and *****p* < 0.0001.

**Figure S4.** **(A)** C57BL/6 mice were injected subcutaneously in the inguinal region with Gpnmb/CpG, CK5-Gpnmb, K36-GC-NV or CK5-GC-NV at equivalent Gpnmb-Cy5 dose (20 μg per mouse), the fluorescence intensity in the inguinal region was monitored by in-vivo animal imaging over time to monitor local retention kinetics at the injection site (n = 4). **(B)** Representative flow cytometric analysis of CD11c^+^CD80^+^ and CD11c^+^CD86^+^ DCs in inguinal lymph nodes (iLNs) of mice (n = 4). (**C**) Representative flow cytometry plots and statistical quantification of p16-expressing senescent dendritic cells (CD11c⁺ p16⁺) in the inguinal lymph nodes (iLNs) harvested from 63-week-old HFD-fed mice across the indicated treatment groups (n = 4). (**D**) Representative flow cytometry plots and corresponding quantitative analysis of Gpnmb-presenting dendritic cells (CD11c⁺ Gpnmb⁺) in the iLNs of mice following immunization with different vaccine formulations (n = 4). **(E)** Representative flow cytometric analysis and statistical data of CD8^+^CD69^+^ and CD8^+^CD25^+^ T lymphocytes in iLNs of mice (n = 4). **(F)** Representative flow cytometric analysis of CD8^+^IFNγ^+^ and CD8^+^GzmB^+^T lymphocytes in iLNs of mice (n = 4). **(G)** Validation of the in vitro surrogate target model, demonstrating the successful induction of senescence in the 3T3-L1 preadipocyte cell line using D-galactose. **(H)** Phenotypic analysis of seno-antigen expression, confirming substantially upregulated Gpnmb levels in the D-galactose-induced senescent 3T3-L1 cells. **(I)** Lactate dehydrogenase (LDH) release assay evaluating in vitro cytotoxicity. The data show the specific cytolytic activity of lymph node-derived activated CD8^+^ T cells from CK5-GC-NV-immunized mice compared to control groups when co-cultured with Gpnmb^+^ senescent 3T3-L1 target cells. **(J)** Flow-cytometric gating hierarchy identifying viable singlet CD8⁺ T cells, followed by CD44/CD62L resolution of central memory (Tcm: CD8^+^CD44^+^CD62^+^) and effector memory (Tem: CD8^+^CD44^+^CD62^-^) subsets. Representative flow plots and quantification across treatment groups at week 70 (n = 4)

**Figure S5.** **(A)** Quantification of SA-β-gal activity in eVAS (n = 4). **(B)** Quantification of Gpnmb, p16, p21, and p53 protein expression based on Western blot analysis (n= 3). (**C)** Adipose tissue was enzymatically dissociated into single-cell suspensions, and CD31⁻Gpnmb⁺ cells were analyzed by flow cytometry (n = 4). **(D)** Representative hematoxylin and eosin (H&E) staining of visceral adipose tissue and quantification of adipocyte cross‑sectional area (>150 adipocytes per animal; n = 10). **(E)** Serum levels of senescence-associated secretory phenotype (SASP) factors were measured by ELISA in the indicated groups of mice (n = 4). (**F)** Average weekly food intake of HFD-induced aged mice post-immunization in the indicated groups (n = 6).

**Figure S6.** **(A)** The statistical data of CD11c^+^ CD80^+^ and CD11c^+^ CD86^+^ DCs in LNs of mice (n = 4). **(B)** Representative flow cytometric analysis of CD8^+^CD69^+^ and CD8^+^CD25^+^ T lymphocytes and statistical data in LNs of mice (n = 4). **(C)** Representative flow cytometric analysis of CD8^+^ IFNγ^+^ and CD8^+^ GzmB^+^T lymphocytes and statistical data in LNs of mice (n = 4). **(D)** Quantitative analysis of p16, p21, p53, and Gpnmb protein expression levels as determined by Western blotting (n= 3). (**E)** Statistical data in CD31^-^Gpnmb^+^ adipocytes of mice (n = 4). **(F)** The quantification of adipocyte cross-sectional area (>150 adipocytes per animal) (n= 10). **(G)** ELISA was used to quantify the levels of SASP factors in the serum of mice from various groups (n= 4). **(H)** Weekly body weight changes and food intake of naturally aged mice following subcutaneous immunization across the indicated treatment groups (n= 6). **(I)** Histological safety evaluation of major organs in naturally aged mice. Representative hematoxylin and eosin (H&E) staining images of the heart, liver, spleen, lung, and kidney harvested from naturally aged mice in the indicated treatment groups (PBS and CK5-GC-NV). All data are expressed as mean ± SD, using GraphPad software for statistical analysis, and the differences were assessed using one-way ANOVA. **p* <0.05, ***p* <0.01, ****p* <0.001 and *****p* < 0.0001.

**Figure S7.** **(A)** Quantitative analysis of p16, p21, p53, and Gpnmb protein expression levels as determined by Western blotting (n= 3). (**B)** Statistical data in CD31^-^Gpnmb^+^ adipocytes of mice (n = 4).
